# N6-methyladenine regulator-mediated RNA methylation modification patterns in immune microenvironment regulation of osteoarthritis

**DOI:** 10.3389/fgene.2023.1113515

**Published:** 2023-01-26

**Authors:** Yong Gu, Zhengming Wang, Rui Wang, Yunshang Yang, Peijian Tong, Shuaijie Lv, Long Xiao, Zhirong Wang

**Affiliations:** ^1^ Translational Medical Innovation Center, Zhangjiagang TCM Hospital Affiliated to Nanjing University of Chinese Medicine, Zhangjiagang, China; ^2^ Department of Orthopedics, Zhangjiagang TCM Hospital Affiliated to Nanjing University of Chinese Medicine, Zhangjiagang, China; ^3^ Shi’s Center of Orthopedics and Traumatology, Shuguang Hospital Affiliated to Shanghai University of Traditional Chinese Medicine, Shanghai, China; ^4^ Institute of Traumatology and Orthopedics, Shanghai Academy of Traditional Chinese Medicine, Shanghai, China; ^5^ The First Affiliated Hospital of Zhejiang Chinese Medical University, Zhejiang Provincial Hospital of Chinese Medicine, Hangzhou, China

**Keywords:** N6-methyladenine (m^6^A), osteoarthritis, methylation, immune, microenvironment

## Abstract

**Background:** Osteoarthritis is a common chronic degenerative disease, and recently, an increasing number of studies have shown that immunity plays an important role in the progression of osteoarthritis, which is exacerbated by local inflammation. The role of N6-methyladenine (m^6^A) modification in immunity is being explored. However, the role of m^6^A modification in regulating the immune microenvironment of osteoarthritis remains unknown. In this study, we sought to discuss the association between the N6-methyladenine (m^6^A) modification and the immune microenvironment of osteoarthritis.

**Methods:** First, the data and gene expression profiles of 139 samples, including 33 healthy samples and 106 osteoarthritis samples, were obtained from the Genetics osteoARthritis and Progression (GARP) study. Then the differences in m^6^A regulators between healthy individuals and osteoarthritis patients were analyzed. The correlation between m^6^A regulators and immune characteristics was also investigated by single-sample gene set enrichment analysis (ssGSEA). Principal component analysis (PCA), Gene Set Variation Analysis (GSVA) enrichment analysis, weighted gene coexpression network analysis (WGCNA), and Associated R packages were used to identify the m^6^A phenotype and its biological functions.

**Results:** A total of 23 m^6^A regulators were involved in this study. We found a close correlation between most m^6^A regulators in all samples as well as in osteoarthritis samples. VIRMA and LRPPRC were the most highly correlated m^6^A regulators and showed a positive correlation, whereas VIRMA and RBM15B were the most negatively correlated. M^6^A regulators are associated with osteoarthritis immune characteristics. For example, MDSC cell abundance was strongly correlated with RBM15B and HNRNPC. Meanwhile, RBM15B and HNRNPC were important effectors of natural killer cell immune responses. IGFBP3 is an important regulator of cytolytic activity immune function. We performed an unsupervised consensus cluster analysis of the osteoarthritis samples based on the expression of 23 m^6^A regulators. Three different m^6^A subtypes of osteoarthritis were identified, including 27 samples in subtype C1, 21 samples in subtype C2, and 58 samples in subtype C3. Different m^6^A subtypes have unique biological pathways and play different roles in the immune microenvironment of osteoarthritis.

**Conclusion:** The m^6^A modification plays a crucial role in the diversity and complexity of the immune microenvironment in osteoarthritis.

## 1 Introduction

Osteoarthritis (OA) is a common chronic degenerative disease that is characterized by joint pain, swelling, and limited activity, resulting in decreased activity and dysfunction of elderly individuals (Abramoff and Caldera, 2020). Patients may endure severe pain with decreased joint mobility, resulting in rising healthcare system costs and decreased work productivity. It was thought in the past that OA is simply produced by mechanical wear and tear and that its mechanism was an imbalance in joint biomechanics (Vincent, 2013). Recently, OA has been understood to result from a complex interplay of local and systemic factors. An increasing number of studies have demonstrated that immune cell infiltration plays an important role in the progression of OA and that local inflammation further aggravates the disease process (Moradi et al., 2015; Rosshirt et al., 2019). The body’s natural wound healing response is manifested in osteoarthritic joints, and there is growing interest in how immunity influences disease initiation and progression (Daghestani and Kraus, 2015). Therefore, immunomodulation in osteoarthritis may be key to the new pathological mechanisms behind it and may shed some light on the discovery of novel immunotherapies for osteoarthritis.

Currently, an increasing number of studies are revealing a novel mode of inheritance, epigenetics, which is based on changes in the expression levels of genes caused by non-genetic sequence alterations (Harvey et al., 2018). Among them, as the third layer of epigenetics, more than 150 RNA modifications have been identified, including N1-methyladenosine (m^1^A), N6-methyladenine (m^6^A), 5-methylcytosine (m^5^C), and 7-methylguanosine (m^7^G). Notably, m^6^A is the most abundant form and has received substantial attention (Patil et al., 2016). It is a dynamic and reversible RNA modification that is involved in a wide range of biological and pathological processes, such as cancer progression and inflammation (Lan et al., 2019; Zong et al., 2019). m^6^A is the most common chemical modification of eukaryotic mRNA and is important in the regulation of mRNA stability, splicing, and translation (Cao et al., 2016). Its regulatory proteins include writers (METTL3, METTL14, WTAP, etc.), erasers (FTO, ALKBH5, etc.), and readers (YTHDF1, YTHDF2, YTHDF3, etc.) (Yang et al., 2018).

Recent studies have identified that m^6^A modification can regulate various aspects of immune function, including immune recognition, activation of innate and adaptive immune responses, and cell fate decisions (Shulman and Stern-Ginossar, 2020). Despite increasing evidence for the regulatory role of m^6^A in immune responses, current studies focusing on the role of m^6^A modification in the immune-related pathogenesis of osteoarthritis are still lacking. Existing studies have mainly focused on METTL3 and FTO (Liu et al., 2019; arc et al., 2012; Panoutsopoulou et al., 2014). The correlation between m^6^A regulators and osteoarthritis remains elusive and requires further exploration. In-depth investigation of immune dysregulation between normal samples and osteoarthritis samples as well as among the various subtypes of osteoarthritis and how m^6^A regulators act on these changes may shed light on osteoarthritis pathogenesis from a new perspective.

However, previous studies have been limited to a few m^6^A regulators due to technical limitations. In this study, we systematically evaluated the modification patterns of m^6^A regulators in osteoarthritis, which furthers our understanding of the immune microenvironment in osteoarthritis. We found that the classification model based on m^6^A regulators could distinguish osteoarthritis samples from healthy samples. There was a high degree of coordination and correlation between m^6^A regulators and infiltrating immune cells, immune responses, and immune functions in osteoarthritis. We identified 3 distinct m^6^A-modified subtypes where different immune characteristics were observed, and we compared the biological functions of these subtypes. In addition, we studied 1175 m^6^A phenotype-related genes and their biological functions. In conclusion, the effect of m^6^A modification on the immune microenvironment of osteoarthritis cannot be ignored.

## 2 Materials and methods

### 2.1 Dataset sources and preprocessing

The data used in this study consisted of 139 samples, including 33 healthy samples and 106 osteoarthritis samples. These samples were obtained from 139 participants of the Genetics osteoARthritis and Progression (GARP) study, and gene expression profiles were extracted from peripheral blood mononuclear cells (PBMCs) of these participants (The age and gender information are provided in Supplementary Table S1). The sample processing protocol and RNA extraction method were well described in a previous study (Ramos et al., 2014). The dataset was deposited in the Gene Expression Omnibus (GEO) database with the accession number GSE48556. The R/Bioconductor package “GEOquery” (Davis & Meltzer, 2007) was used to extract the GEO dataset, which consisted of the gene expression matrix and clinical features. According to the annotation information of the GPL6947 platform, probe mapping was applied to genes. If multiple probes corresponded to one gene, the average value was taken, and probes corresponding to multiple genes were deleted. Matrix expression values were preprocessed by correction with the “normalizeBetweenArrays” function in the “limma” package (Ritchie et al., 2015).

### 2.2 Alteration analysis of m^6^A regulators between healthy individuals and osteoarthritis patients

These 23 m^6^A regulators involved in the study included 8 writers (METTL3, METTL14, METTL16, WTAP, VIRMA, ZC3H13, RBM15, and RBM15B), 2 erasers (FTO and ALKBH5), and 13 readers (YTHDC1, YTHDC2, YTHDF1, YTHDF2, YTHDF3, HNRNPC, FMR1, LRPPRC, HNRNPA2B1, IGFBP1, IGFBP2, IGFBP3, and RBMX). The expression relationships among the 23 m^6^A regulators were evaluated by Spearman correlation analysis in all samples and osteoarthritis samples. Then, we constructed a correlation network of these 23 m^6^A regulators. The expression differences of the 23 m^6^A regulators between healthy and osteoarthritis samples were compared by the Wilcoxon test. OA-related m^6^A regulators were determined by univariate logistic regression with a cutoff criterion of *p*-value <0.2. Least absolute shrinkage and selection operator (LASSO) regression was used for feature selection and dimension reduction. Multivariate logistic regression was used to develop a m^6^A regulator-associated osteoarthritis classification model and external data sets (Details are provided in Supplementary Table S2) were used for verification. Receiver operating characteristic (ROC) curve analysis was used to evaluate the discriminatory performance of the model signatures.

### 2.3 Correlation between m^6^A regulators and immune characteristics

Single-sample gene set enrichment analysis (ssGSEA) was used to estimate the abundance of specific infiltrating immune cells and the activity of specific immune responses and immune function. It defines an enrichment fraction to express the absolute degree of enrichment of a gene set in each sample (Shen et al., 2019). The immune cell gene set and the immune function gene set were derived from previous studies (Zhang et al., 2020a; Liang et al., 2020). The immune genes and immune response gene sets were obtained from the ImmPort database (http://www.immport.org) (Bhattacharya et al., 2014). The Wilcoxon test was used to compare the abundance of immune cells, immune response, and immune function enrichment scores between healthy and osteoarthritis samples. We analyzed the correlation between the expression of m^6^A regulators and the immune cell fraction, immune response activity, and immune function activity by the Spearman method.

### 2.4 Identification of distinct m^6^A modification patterns by unsupervised clustering

The ConsensusClusterPlus package was applied to classify disease samples into distinct subtypes based on the expression of 23 m^6^A regulators (Wilkerson and Hayes, 2010). This is an unsupervised clustering analysis method. The Euclidean distance was utilized to calculate the similarity distance between samples, and the K-means algorithm was used to evaluate cluster numbers and robustness (Hartigan and Wong, 2013). The maximum cluster number was set to be 9. Eighty percent of the samples were sampled by the resampling scheme, and resampling was conducted 1000 times. The final cluster number was determined by the consensus matrix and the cluster consensus score (>0.8). Principal component analysis (PCA) was used to further verify the distinct modification patterns of 23 m^6^A regulators.

### 2.5 Immune characteristics and biological enrichment analysis of distinct m^6^A modification subtypes

We compared the differences in the immune cell fraction, immune response activity, and immune function activity among the m^6^A subtypes by the Kruskal test. To investigate the differences in biological functions and processes between m^6^A modification patterns, Gene Set Variation Analysis (GSVA) enrichment analysis was applied by the “GSVA” package. GSVA, known as gene set variant analysis, is a non-parametric unsupervised analysis method that transforms the expression matrix of genes across different samples into a pathway activation score matrix and evaluates whether different biological pathways are enriched across samples (Hanzelmann et al., 2013). The HALLMARKS pathway and KEGG pathway are two commonly used pathway gene sets. From the MSigDB database (http://www.gsea-msigdb.org/gsea/msigdb), the “h.all.v7.4.symbols” and “c2.cp.kegg.v7.4.symbols” gene sets were downloaded for running the GSVA analysis. Pathway activation scores were compared between the two groups by the R package “limma”, and adjusted *p*-values less than 0.05 were considered statistically significant.

### 2.6 Identification of m^6^A phenotype-related genes

To identify genes mediated by m^6^A regulators, differentially expressed genes (DEGs) between distinct m^6^A phenotypes were analyzed by the empirical Bayesian method of the “limma” R package, and the cutoff criterion for screening DEGs were set as adjusted *p*-value <0.01. The biological functions of m^6^A phenotype-related genes were analyzed by GO and KEGG enrichment analysis using the R package “clusterProfiler” (Yu et al., 2012). WGCNA (weighted gene coexpression network analysis) was used to identify the modification pattern-related gene modules through the “WGCNA” package (Langfelder and Horvath, 2008).

## 3 Results

### 3.1 Landscape of m^6^A regulators between healthy and osteoarthritis samples

**FIGURE 1:**
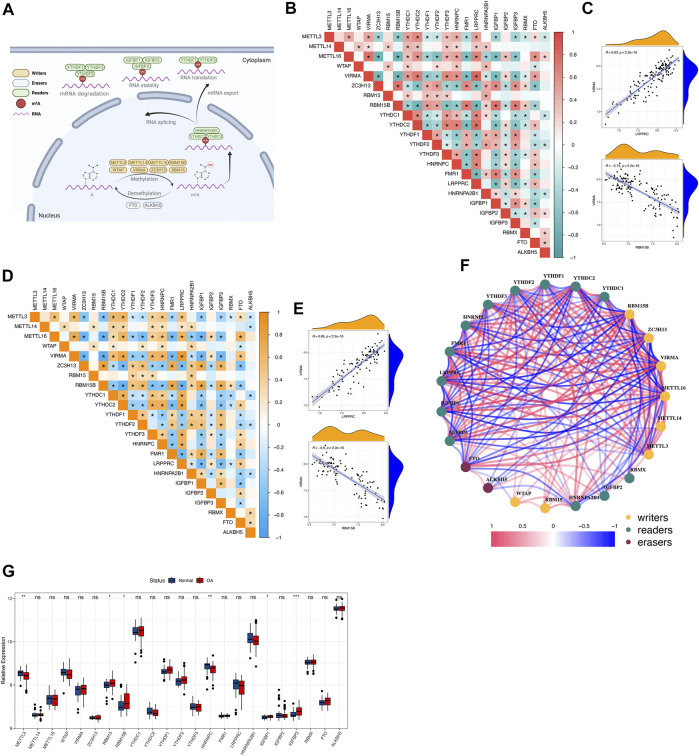
The landscape of m^6^A RNA methylation regulators in osteoarthritis **(A)** Overview of the dynamic reversible process of m^6^A RNA methylation modification regulated by “writers”, “erasers” and “readers” in osteoarthritis and their potential biological functions for RNA. **(B,C)** The correlation of the expression of 23 m^6^A regulators in all samples. Red indicates a positive correlation, and green indicates a negative correlation. Two scatter plots showed the two most relevant sets of m^6^A regulators: VIRMA and LRPPRC were the most positively correlated and VIRMA and RBM15B were the most negatively correlated. Above * means *p* < 0.05. **(D,E)** The correlation of the expression of 23 m^6^A regulators in osteoarthritis samples. Orange indicates a positive correlation, and blue indicates a negative correlation. Two scatter plots showed the two most relevant sets of m^6^A regulators: VIRMA and LRPPRC were the most positively correlated and VIRMA and RBM15B were the most negatively correlated. Above * means *p* < 0.05. **(F)** The regulatory network of 23 m^6^A regulators in osteoarthritis: red indicates a positive correlation, and blue indicates a negative correlation. **(G,H)** The boxplot and heatmap show the expression of 23 m^6^A regulators between healthy and osteoarthritis samples. Above * indicates *p* < 0.05, ** indicates *p* < 0.01, *** indicates *p* < 0.001, and ns indicates that the difference was not statistically significant.

A total of 23 m^6^A regulators were involved in this study, including 8 writers, 13 readers, and 2 erasers. An overview of m^6^A regulators and their functions was given (Figure 1A). By analyzing the transcriptome matrix, we found a close correlation between most m^6^A regulators in all samples as well as in osteoarthritis samples (Figures 1B, D). This illustrated that these regulators influenced each other, and this correlation was approximately the same across all samples as well as across disease samples. Among them, VIRMA and LRPPRC were the most highly correlated m^6^A regulators expressed in all samples and osteoarthritis samples and showed a positive correlation, whereas VIRMA and RBM15B were the most negatively correlated (Figures 1C, E). These suggested that they function together. We constructed a correlation network of m^6^A regulators in osteoarthritis (Figure 1F), again verifying that they generally function as a group. Differential expression analysis identified 6 m^6^A regulators with altered expression. These factors with altered expression were distributed among the writers and readers, whereas the erasers did not change significantly, suggesting that they might not play an important role in osteoarthritis independently (Figure 1G).

### 3.2 m^6^A regulators contribute to the osteoarthritis process

**FIGURE 2:**
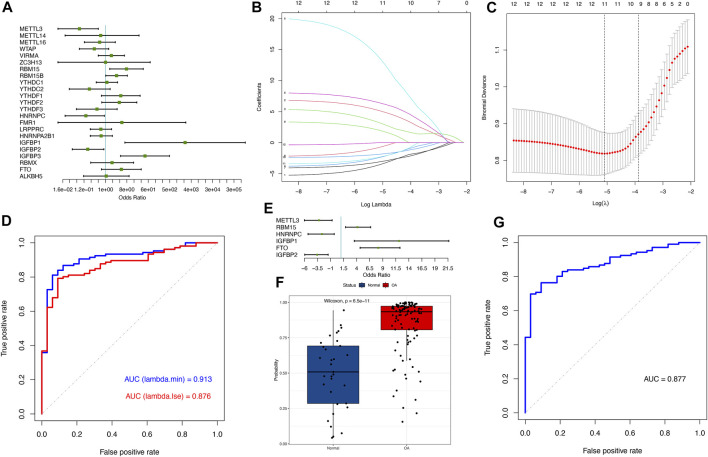
m^6^A regulators have profound effects on the osteoarthritis process. **(A)** Univariate logistic regression presenting the relationship between m^6^A regulators and osteoarthritis revealed 12 OA-related m^6^A regulators (*p* < 0.2). **(B)** LASSO coefficient distribution of 12 OA-associated m^6^A regulators. **(C)** Cross-validation for tuning parameter selection in the LASSO regression. For each *λ* value, around the mean of the target parameter shown by the red dot, we obtained the confidence interval of one target parameter. Two dashed lines indicate two special *λ* values: lambda. min was referred to in all *λ* values, the one giving the minimum target parametric mean, and lambda.1se refers to the one that gave the simplest model with a variance in the lambda. min *λ* value. **(D)** The ROC curve illustrated that the LASSO model, which includes 12 m^6^A regulators according to MSE, has better performance in distinguishing between normal and osteoarthritis samples than 1-SE, with an AUC value of 0.913. **(E)** Multivariate logistic regression presenting the relationship between m^6^A regulators and osteoarthritis revealed 6 osteoarthritis-related m^6^A regulators (*p* < 0.05). **(F)** Boxplot showing the probability difference between normal and osteoarthritis samples, where osteoarthritis had a much higher probability score than normal samples. **(G)** The ROC curve depicting that the classification model based on 6 m^6^A regulators has good prediction performance.

We employed a series of statistical algorithms to explore the impact of m^6^A modification on osteoarthritis pathogenesis. We found that 12 m^6^A regulators were associated with osteoarthritis by univariate logistic regression (Figure 2A; see Supplementary Table S3 in the Supplementary Material). LASSO regression was performed on 12 m^6^A regulators for feature selection and dimensionality reduction to exclude non-significant regulators (Figures 2B, C). We found that all 12 m^6^A regulators were essential for osteoarthritis. When lambda with the minimum squared error (MSE) was employed, 12 variables were obtained, and 8 were obtained when the one-fold standard error (1-SE) was chosen. The ROC curve indicated the higher precision of the former 12 regulators in distinguishing disease from the normal group (AUC = 0.913) (Figure 2D). Multivariate logistic regression was performed to develop a categorical model to discriminate between normal and osteoarthritis samples (Figure 2E; see Supplementary Table S4 in the Supplementary Material). The model consisted of 6 m^6^A regulators and discriminated well between healthy and osteoarthritis samples based on predicted probability values, where the probability scores of osteoarthritis were significantly higher than those of healthy samples (Figure 2F). The ROC curve illustrated the excellent performance of the 6 m^6^A regulators in classifying health and osteoarthritis, indicating their diagnostic value for OA (Figure 2G). We found that our model based on the expression of 6 m^6^A regulators had excellent diagnostic performance in other data sets (Supplementary Figure S1).

### 3.3 m^6^A regulators are associated with osteoarthritis immune characteristics

**FIGURE 3:**
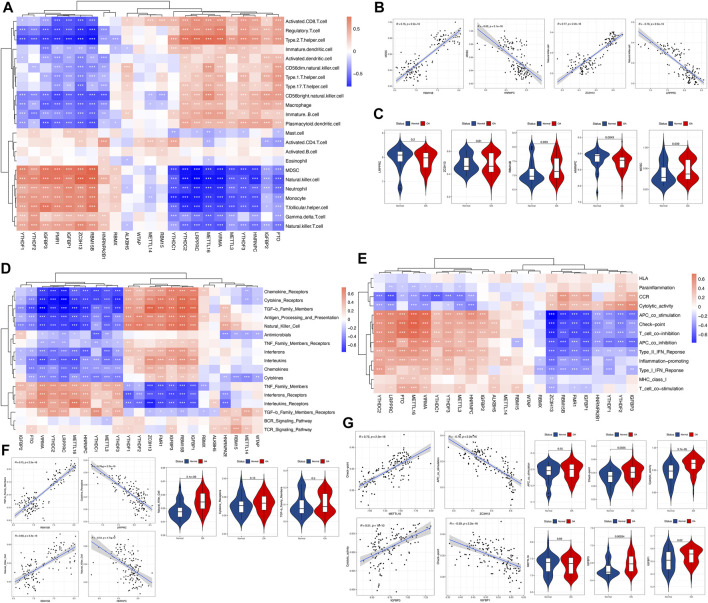
Correlations between the immune cell infiltration fraction, immune response gene sets, immune function gene sets, and m^6^A regulators. **(A)** Heatmap demonstrating the correlations between each immune infiltration cell type and each m^6^A regulator. Red indicates a positive correlation, and blue indicates a negative correlation. **(B,C)** Scatterplot demonstrating the correlations between the dysregulated immune cell fraction and the m^6^A regulator. The fraction or expression status is presented as a violin plot, indicating that there were more MDSCs, higher expression of RBM15B, and lower expression of HNRNPC in osteoarthritis. **(D,F)** Heatmap demonstrating the correlations between each immune response gene set and each m^6^A regulator. For dysregulated m^6^A regulators, the most positively correlated pair was natural killer cell-RBM15B, and the most negatively correlated pair was natural killer cell-HNRNPC. There was a more active natural killer cell reaction in osteoarthritis, as presented by the violin plot. **(E,G)** Heatmap demonstrating the correlations between each immune function gene set and each m^6^A regulator. For dysregulated m^6^A regulators, pairs with a stronger positive correlation were cytolytic activity-IGFBP3, whereas a negative correlation was found for check-point-IGFBP1, and there were stronger check-point and cytolytic activity functions activated in osteoarthritis, as presented by the violin plot.

To investigate the biological behaviors between m^6^A regulators and the immune microenvironment, we performed correlation analyses between m^6^A regulators and immune infiltration cells, immune responses, and immune functions. First, the results revealed differences in the abundance of 23 infiltrating immune cells between healthy and osteoarthritis samples (See Supplementary Figure S2 in the Supplementary Material for comprehensive image analysis). Some immune cells changed in osteoarthritis, such as myeloid-derived suppressor cells (MDSCs), T follicular helper cells, type 1 T helper cells and type 17 T helper cells, involving innate immunity and adaptive immunity. Correlation analysis identified that m^6^A regulators were closely associated with these immune cells (Figure 3A). For example, the MDSC cell abundance was positively correlated with RBM15B and negatively correlated with HNRNPC (Figures 3B, C). This showed that infiltrating MDSCs were increased in osteoarthritis, which was closely related to the expression of RBM15B and HNRNPC. Similarly, we analyzed the immune response in osteoarthritis. The differences in the activity of each immunoreaction gene set between the healthy and osteoarthritis samples are presented (See Supplementary Figure S3 in the Supplementary Material for comprehensive image analysis). Several immune responses were increased in osteoarthritis, such as natural killer cell activity and TNF family members receptors. Natural killer cell activity was positively correlated with RBM15B, but it was negatively correlated with HNRNPC (Figures 3D, F). This suggested that RBM15B and HNRNPC played important roles in the natural killer cell response of osteoarthritis. We observed that RBM15B had a positive regulatory effect on multiple immune responses, while HNRNPC showed a negative regulatory effect. We also investigated the active state of immune function, in which half of the immune functions were altered in patients with osteoarthritis (See Supplementary Figure S4 in the Supplementary Material for comprehensive image analysis). For example, the check-point, cytolytic activity, and T-cell costimulation scores were higher in osteoarthritis. We found that METTL16 and ZC3H13 were positively and negatively correlated with most immune functions, respectively (Figures 3E, G). Check-point-METTL16 was the most positively correlated pair, and the most negatively correlated pair was APC costimulation-ZC3H13 (Figure 3G). However, for dysregulated m^6^A regulators, pairs with a stronger positive correlation were cytolytic activity-IGFBP3, whereas a negative correlation was with check-point-IGFBP1 (Figure 3G).

### 3.4 Patterns of m^6^A methylation modification mediated by 23 regulators in osteoarthritis

**FIGURE 4:**
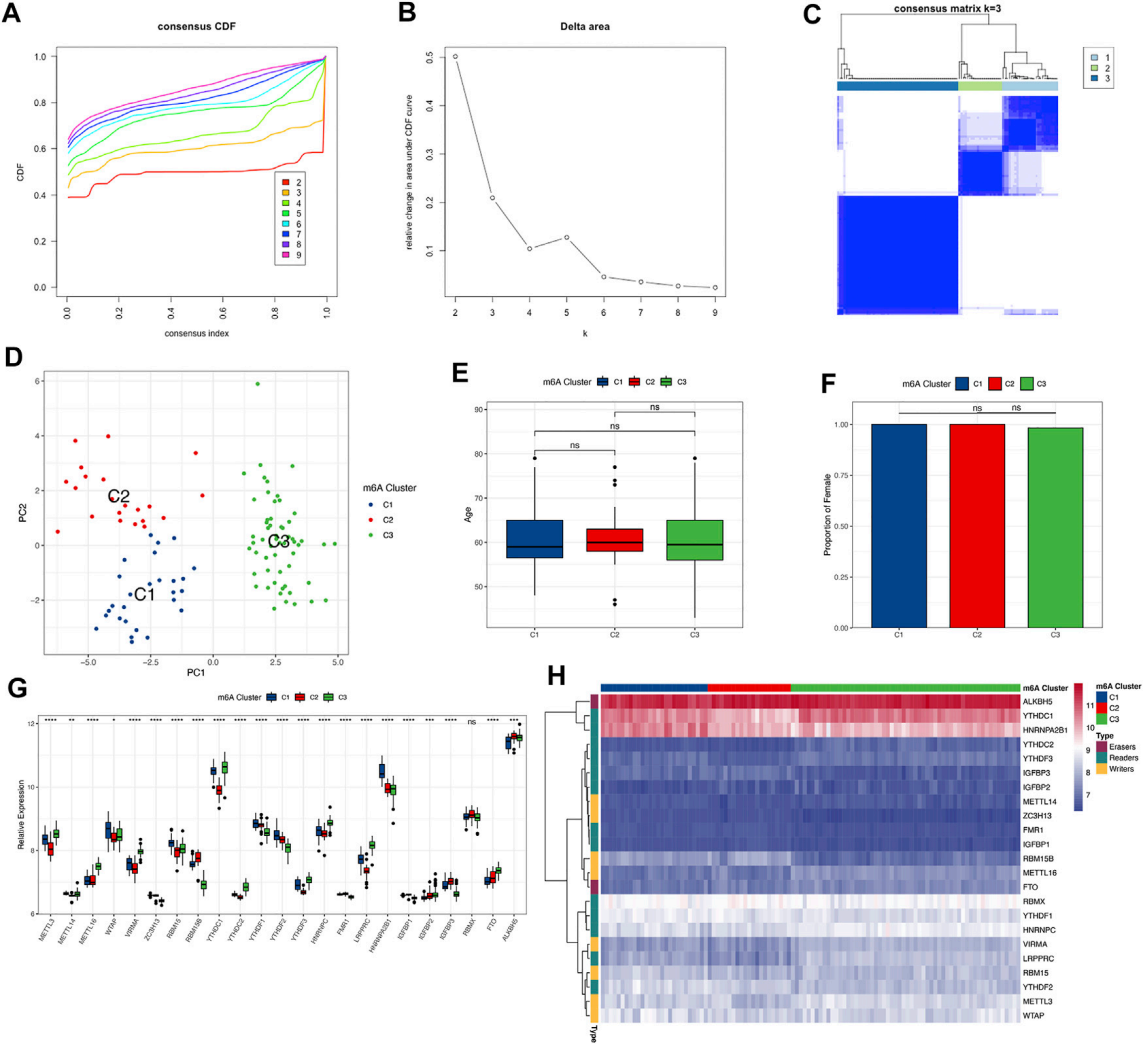
Identification of subtypes by unsupervised clustering based on the expression of 23 m^6^A regulators. **(A)** Consensus clustering cumulative distribution function (CDF) for k = 2–9. **(B)** Relative change in the area under the CDF curve for k = 2–9. **(C)** Heatmap of the consensus matrix for osteoarthritis samples. **(D)** PCA of the transcriptome profiles of 3 m^6^A subtypes, showing a remarkable difference in the transcriptome between different modification patterns. **(E,F)** Comparison of age and gender. The boxplot illustrated the association of age with the 3 subtypes. The bar plot illustrates the association of age with the 3 subtypes. Above ns means the difference was not statistically significant. **(G)** Expression differences of 23 m^6^A regulators among the 3 m^6^A subtypes. Above * indicates *p* < 0.05, ** indicates *p* < 0.01, *** indicates *p* < 0.001, **** indicates *p* < 0.0001, and ns indicates that the difference was not statistically significant. **(H)** Heatmap of the expression status of 23 m^6^A regulators in the 3 subtypes with unsupervised clustering.

To investigate the m^6^A modification patterns in osteoarthritis, we performed an unsupervised consensus cluster analysis of the osteoarthritis samples based on the expression of 23 m^6^A regulators. Three different m^6^A subtypes of osteoarthritis were identified, including 27 samples in subtype C1, 21 samples in subtype C2, and 58 samples in subtype C3 (Figures 4A–C). The results of PCA confirmed that the 23 m^6^A regulators could discriminate the 3 subtypes well (Figure 4D). The 3 distinct modification patterns differed from the current osteoarthritis classification, with no significant differences in clinical features between the different modification patterns, such as sex and age (Figures 4E, F). Except for RBMX, all m^6^A regulators showed significant differences in their expression among the 3 m^6^A subtypes (Figure 4G). The 23 m^6^A regulators could still be divided into 3 parts according to their expression levels (Figure 4H), verifying the diversity of m^6^A modification patterns in osteoarthritis.

### 3.5 Immune characteristics of 3 distinct m^6^A subtypes

**FIGURE 5:**
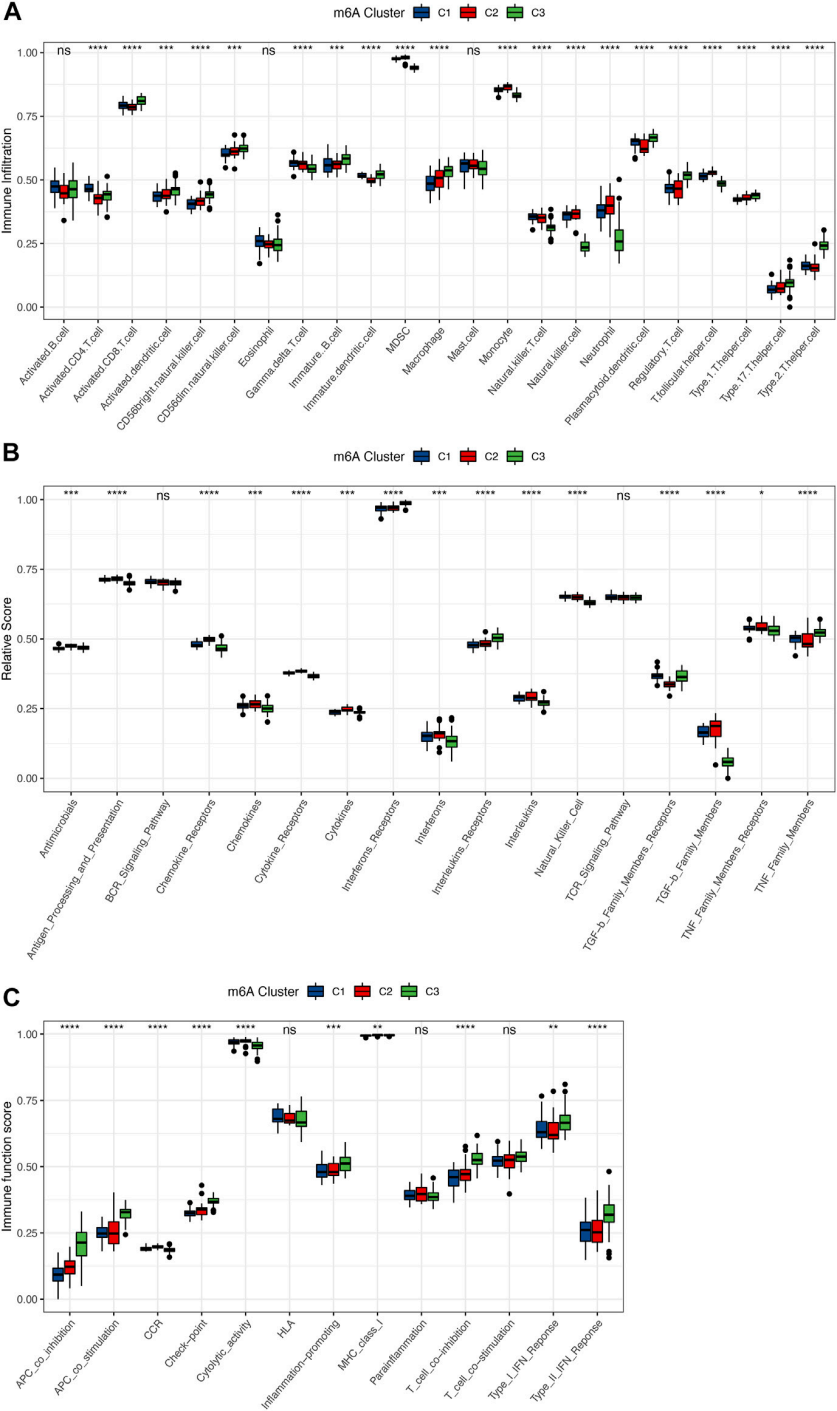
Immune microenvironment characteristics among 3 distinct m^6^A subtypes. **(A)** Differences in the abundance of each immune cell infiltration in 3 m^6^A subtypes. **(B)** Activity differences of each immune response gene set in 3 m^6^A subtypes. **(C)** Expression differences of each immune function gene set in 3 m^6^A subtypes. Above * indicates *p* < 0.05, ** indicates *p* < 0.01, *** indicates *p* < 0.001, **** indicates *p* < 0.0001, and ns indicates that the difference was not statistically significant.

To determine the differences in immune microenvironment features among these different m^6^A modification patterns, infiltrating immune cells, immune response gene sets, and immune function gene sets were assessed, and we found that the immune features were different among the three groups. The vast majority of the immune cells were distinct in the 3 patterns (Figure 5A). Subtype C3 had relatively higher infiltrating immune cells than subtypes C1 and C2, and the immune cell infiltration status of C1 was closer to that of C2. Subtype C3 had higher levels of activated CD8 T Cells, CD56bright natural killer cells, immune B Cells and macrophages, whereas monocytes, natural killer cells and MDSCs were enriched in subtype C2. A similar pattern in terms of immunoreactivity was observed, with more complex results. The immunoreactivity of subtype C3 differed from those of subtypes C1 and C2, with the status of C1 and C2 more similar, whereas the immunoreactivity of C2 was more active. For example, chemokines, cytokine receptor, and cytokines were more active in subtype C2, while interferon receptors and interleukins were more active in subtype C3, and TGF-b family members were much lower than in C1 and C2 (Figure 5B). Similar trends were also observed in the immune function scores (Figure 5C). The differences in terms of immune function were greater in subtype C3 than in C1 and C2. These results suggested that m^6^A modification of subtype C3 mediated a unique immune inflammatory response that was distinguished from subtypes C1 and C2, whereas subtypes C1 and C2 also mediated distinct immune responses. The above results once again demonstrated that m^6^A methylation modification had an important regulatory effect on the formation of different immune microenvironments in osteoarthritis.

### 3.6 Biological properties of the 3 m^6^A modification patterns

**FIGURE 6:**
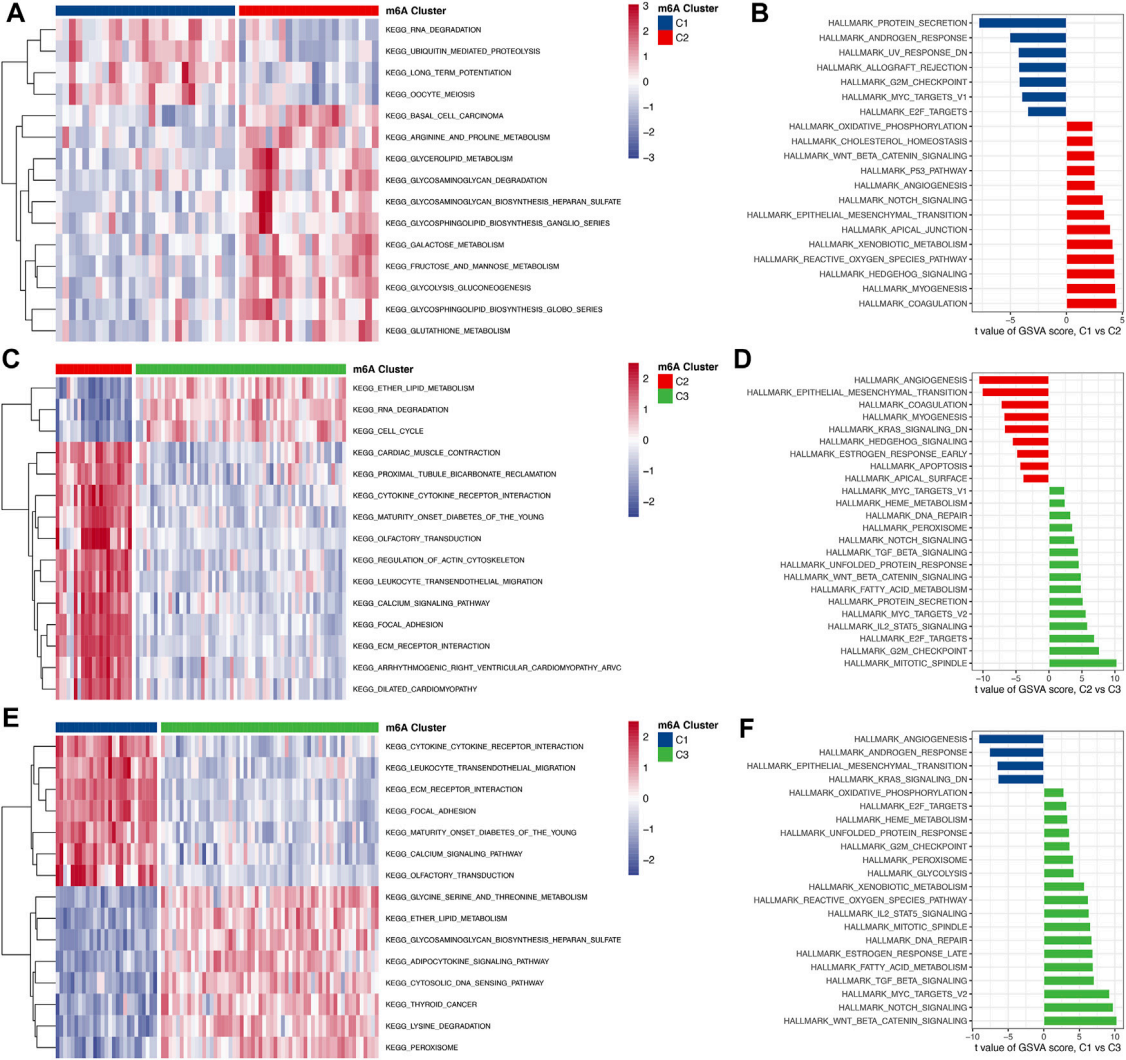
The biological function characteristics among the 3 m^6^A subtypes. **(A,B)** Differences in GSVA enrichment scores for the KEGG pathway and HALLMARK pathway between m^6^A cluster 1 and cluster 2 (A for the KEGG pathway and B for the HALLMARK pathway). **(C,D)** Differences in GSVA enrichment scores for the KEGG pathway and HALLMARK pathway between m^6^A cluster 2 and cluster 3 (A for the KEGG pathway and B for the HALLMARK pathway). **(E,F)** Differences in GSVA enrichment scores for the KEGG pathway and HALLMARK pathway between m^6^A cluster 1 and cluster 3 (A for the KEGG pathway and B for the HALLMARK pathway).

**FIGURE 7:**
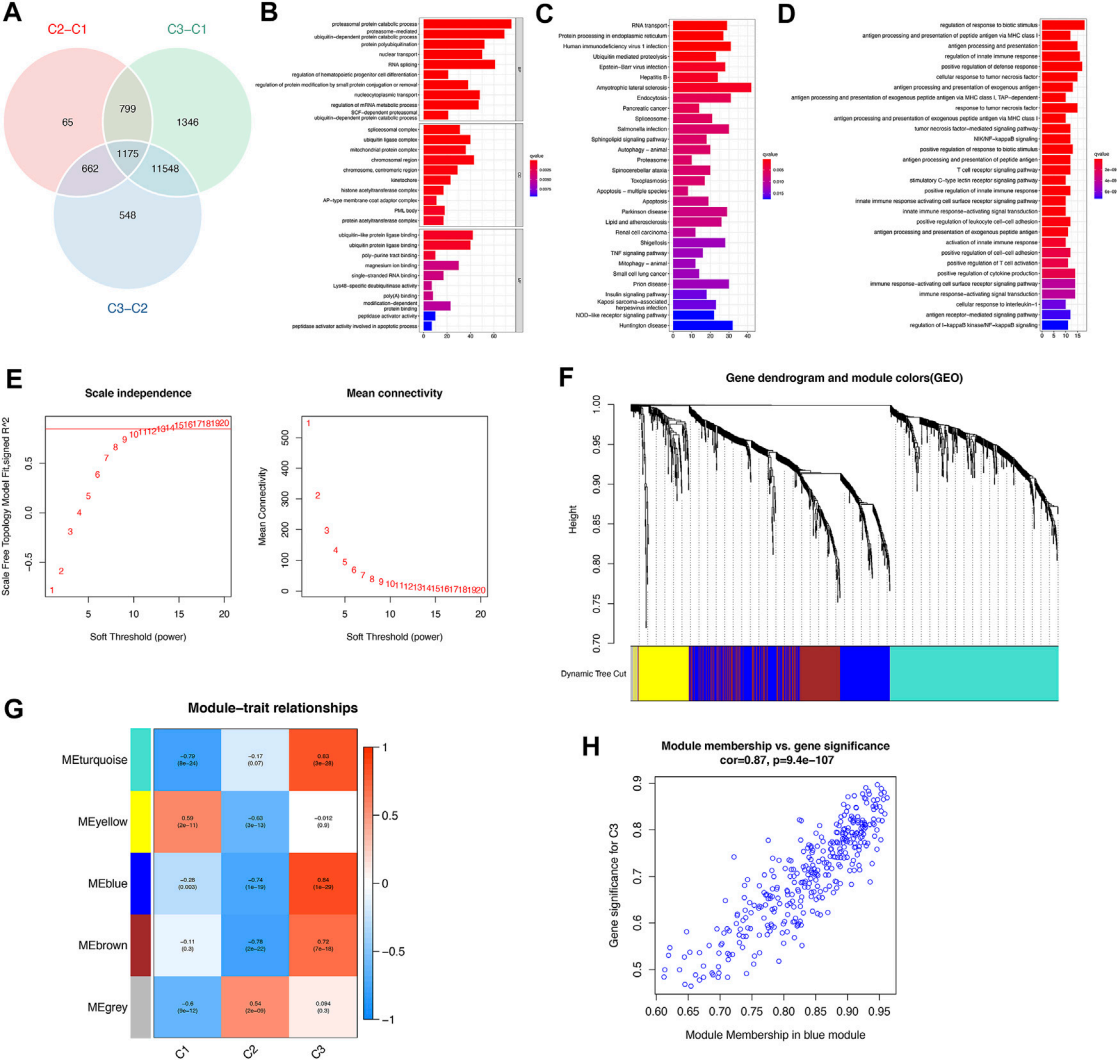
Identification and functional analysis of m^6^A phenotype-related genes in osteoarthritis. **(A)** Venn diagram of 1175 m^6^A phenotype-related genes. **(B)** GO enrichment analysis revealed the biological function characteristics of m^6^A phenotype-related genes. **(C)** KEGG enrichment analysis revealed the biological signaling pathways of m^6^A phenotype-related genes. **(D)** GO-BP enrichment results revealed the biological processes (BPs) of m^6^A modification-mediated immune genes. **(E)** Analysis of the scale-free topology model fit index and the mean connectivity for various soft-thresholding powers. **(F)** Gene dendrogram obtained by average linkage hierarchical clustering. The color row underneath the dendrogram shows the module assignment determined by the dynamic tree cut, in which 5 modules were identified. **(G)** Heatmap of the correlation between characteristic gene modules and 3 distinct m^6^A subtypes: red indicates a positive correlation, and blue indicates a negative correlation. **(H)** The scatterplot of gene significance for m^6^A subtype C3 *vs*. module membership in the blue module. The gene significance and module membership exhibited highly significant correlations, implying that the hub genes of the blue module also tended to be highly correlated with subtype C3.

To investigate the biological responses in the 3 m^6^A subtypes, we compared KEGG pathways and HALLMARKS pathways between each of them and applied GSVA enrichment analysis to evaluate the activation status of biological pathways. Compared with subtypes C1 and C3, subtype C2 had more enriched pathways, such as the ECM receptor interaction, calcium signaling, cytokine to cytokine interaction receptor, and leukocyte transendothelial migration pathways (Figures 6A–D). Subtypes C1 and C3 had almost the same number of enriched pathways compared with each other (Figures 6E, F). To further understand the molecular mechanisms by which genes were involved in the regulation mediated by m^6^A regulators, we obtained differential gene intersections between m^6^A phenotypes to obtain phenotype-related genes. As a result, a total of 1175 m^6^A phenotype-related genes were obtained (Figure 7A), and GO enrichment analysis revealed that they were mainly involved in RNA splicing, nuclear transport, and regulation of mRNA metabolic processes (Figure 7B).

KEGG enrichment analysis showed that signaling pathways mainly involved RNA transport, protein processing in the endoplasmic reticulum, the TNF signaling pathway, and the NOD−like receptor signaling pathway (Figure 7C). We extracted 57 immune genes from these m^6^A phenotype-related genes (See Supplementary Table S5 in the Supplementary Material), of which the enriched biological processes were remarkably related to the regulation of the response to biotic stimulus, regulation of the innate immune response, cellular response to tumor necrosis factor, and T Cell receptor signaling pathway (Figure 7D). We then constructed a comprehensive gene map associated with m^6^A modification patterns, and WGCNA identified gene-gene modules associated with distinct m^6^A subtypes (Figures 7E, F). Five gene modules were identified, and distinct modification patterns matched their related genes (Figure 7G; see Supplementary Table S6 in the Supplementary Material). For example, genes in the blue module were highly correlated with the m^6^A regulator modification pattern C2 (Figure 7H). These genes were highly correlated not only with their corresponding modules but also with their corresponding subtypes, further illustrating that genes deserve deep exploration. These results might elucidate the gene expression regulatory network mediated by m^6^A regulators.

## 4 Discussion

Osteoarthritis is a chronic degenerative disease with a complex pathological mechanism that has not been clarified thus far. The understanding of this process has gone beyond mechanical wear and tear, in which inflammatory processes and immune responses also exist (Woodell-May and Sommerfeld, 2020). Increasing evidence has confirmed the indispensable role of m^6^A modification in innate and adaptive immune responses (Zheng et al., 2017). To date, studies have been carried out to explore the role of m^6^A in immunity, especially in tumor microenvironment infiltrating cells (Han et al., 2019; Yang et al., 2019; Zhang et al., 2019). Therefore, we believe that similar results can be observed in the regulation of the immune microenvironment of osteoarthritis by m^6^A modification. In this study, we systematically investigated the modification pattern of m^6^A in the immune microenvironment of osteoarthritis. To clarify how m^6^A modification shapes the immune cell infiltration, immune response, immune function, and activation pathway of osteoarthritis, we conducted a series of analyses and obtained the following findings.

First, we found that compared with the normal samples, the expression of some m^6^A regulators was out of balance in osteoarthritis. At the same time, there was a close relationship between the 23 regulators. We constructed a regulatory network of m^6^A regulators, which indicates that m^6^A regulators interact with each other and participate in the development of osteoarthritis. We used a series of multiple statistical approaches to screen out the significant m^6^A regulators involved in osteoarthritis. The disease classification model based on these factors can distinguish healthy and osteoarthritis samples well, which confirms the important role of m^6^A regulators in osteoarthritis. METTL3, HNRNPC, and IGFBP1 may be the most important among the 23 m^6^A regulators, and they are of great significance in multivariate analysis. METTL3 has a functional role in mediating osteoarthritis progression by regulating NF-κB signaling and extracellular matrix (ECM) synthesis in chondrocytes (Liu et al., 2019). It has also been further confirmed that the expression of the m^6^A methylated gene METTL3 is decreased in osteoarthritis and may be involved in osteoarthritis by regulating inflammatory responses (Sang et al., 2021). In addition, studies have shown that in pathological conditions, increased concentrations of IGF-I in joint synovial fluid are accompanied by increased levels of IGFBP-1 and IGFBP-3 (Matsumoto et al., 1996). These results are consistent with our findings in peripheral blood. However, the changes in HNRNPC in osteoarthritis have still not been specifically reported.

Next, we explored the correlations between m^6^A regulators and immune characteristics in osteoarthritis, including scores for infiltrating immune cells, immune response, and immune function. We found that most m^6^A regulators were closely associated with these immune characteristics, implying an important role of m^6^A modification in the regulation of the osteoarthritis immune microenvironment. For example, MDSC abundance was strongly positively correlated with RBM15B and negatively correlated with HNRNPC. MDSCs can inhibit body immune cells to exert regular innate and adaptive immune functions. In the context of innate immunity, MDSCs downregulate the expression of NKG2D by membrane-bound TGF-β, which inhibits the function of NK cells (Li et al., 2009). MDSCs can also induce Treg expansion and promote the negative regulatory effect of Treg on immunity (Serafini et al., 2008). In terms of adaptive immunity, MDSCs can inhibit T Cell immune response responses and proliferation through multiple pathways (Rodríguez and Ochoa, 2008). Studies have found that MDSCs are significantly expanded in arthritic mice and RA patients. The transfer of MDSCs promotes disease progression, and proinflammatory MDSCs with the ability to drive Th17 cell differentiation may be a key pathogenic factor in autoimmune arthritis (Guo et al., 2016). RBM15B is reported to recruit this complex to certain mRNA and lncRNA XIST to promote m^6^A formation (Coker et al., 2020). HNRNPC plays a cancer-promoting role in adrenocortical carcinoma (ACC) progression, and experiments have demonstrated that HNRNPC promotes the proliferation, migration, and invasion of H295R and SW13 cells and influences the immune microenvironment (Xu et al., 2021). These findings may point to an immunoregulatory mechanism of m^6^A in osteoarthritis.

Third, unsupervised clustering of osteoarthritis samples based on the expression profiles of 23 m^6^A regulators identified 3 subtypes with unique m^6^A modification patterns, each with unique immune characteristics. Among them, subtype C3 had more infiltrating immune cells and more active immune functions than subtype C1 and subtype C2, and a portion of the immune response was more active in C3. We confirmed the reliability of phenotypic classification of different m^6^A alterations by contrasting immune properties across the subtypes. The inspiration for this approach stems from a recent high-quality study in which a team used this approach to identify 3 distinct novel m^6^A modification patterns in gastric cancer, gaining a deeper understanding of the tumor microenvironment (Zhang et al., 2020a). Identifying new molecular subtypes will not only unearth new pathogenesis but also enable the development of more precise treatment regimens. For osteoarthritis, Coutinho de Almeida R *et al* performed an unsupervised cluster analysis based on the top 1000 gene expressions deregulated in osteoarthritis, resulting in 2 distinct osteoarthritis subtypes possessing distinct cartilage pathophysiological processes as well as radiological features (Coutinho de Almeida et al., 2021). Thus, the 3 different m^6^A subtypes in osteoarthritis suggest that the m^6^A modification patterns present in peripheral blood can indeed be considered another pathobiology-based classification of osteoarthritis, which is related to the phenotypic features of the disease.

Finally, we identified m^6^A phenotype-associated genes and m^6^A modification subtype-associated gene modules. The expression regulation of these genes and gene sets is affected by m^6^A modification, and revealing their biological functions can help illustrate the pathogenesis of osteoarthritis from the perspective of m^6^A modification. Subtype C3 had more activation in the well-known TGF-β signaling pathway, while decreased NK cell infiltration was seen in subtype C3, and IGFBP1 was downregulated in subtype C3. These results may suggest that IGFBP1, the TGF-β signaling pathway, and NK cells are strongly implicated in osteoarthritis. The results of our study can give many of these similar correlations, and other researchers in the field will be directed to rapidly capture key m^6^A regulators and immune signatures in osteoarthritis. This is one of the most important scientific implications of our study.

Although there is no consensus on the immune characteristics of OA, more and more studies have shown that immune inflammation is closely related to the pain and pathological progress of OA in recent years (Zhang et al., 2020b; Miller et al., 2020; Woodell-May and Sommerfeld, 2020; Li et al., 2022). And immunoengineering is expected to become the next-generation of arthritis treatment method (Klimak et al., 2021). Our study is the first to systematically analyze the relationship between m^6^A modification and the immune microenvironment of osteoarthritis, and we are also the first team to introduce the latest m^6^A mechanisms in osteoarthritis. Through this study, we obtained a wealth of results that can open new directions for studying the immune-related pathogenesis of osteoarthritis from the perspective of m^6^A modification mechanisms. In addition, we confirmed that m^6^A modification is involved in the regulation of the immune microenvironment in osteoarthritis. Current correlative studies of m^6^A regulators in the osteoarthritis field are tenuous, and thus, this osteoarthritis research was seminal. We combined the latest m^6^A modification and immune microenvironment theory to unravel osteoarthritis pathogenesis, largely complementing the gap in osteoarthritis regarding epigenetic modifications, particularly m^6^A modification. This study will motivate more researchers to carry out m^6^A-related research in the field of osteoarthritis, and the numerous results of this research can provide a better direction for them.

However, the study has some drawbacks. Firstly, this study was based on bioinformatics analysis, and many of the results were valid in theory, but their accuracy needs to be verified experimentally. Immune cell fraction were calculated by using currently accepted methods, but single-cell sequencing is still required to obtain the most accurate immune cell count. Secondly, some clinical characteristics were not available, such as Osteoarthritis Research Society International (OARSI) score, visual analog scale (VAS) score, joint range of motion and radiographic staging. All these made it difficult to reveal the correlation between clinical severity or outcome and the diversity of m^6^A modification patterns. We also could not perform the analyses that associate the m^6^A-mediated gene expression regulatory network with the progression of OA. In addition, because of the lack of clinical efficacy data, we failed to reflect the advantages of m^6^A patterns compared with current diagnosis methods and its help for follow-up treatment. We hope to obtain data in the future and analyze them from the perspective of multiomics to obtain more valuable results. Thirdly, it is worth noting that expression level of m^6^A regulators is not identical to m^6^A methylation level and clinical samples are required for methylation level detection. Fourthly, Many studies were limited to gene regulation mediated by m^6^A, but ignored the mechanism of regulating m^6^A precipitation (Yang et al., 2022). For example, aging and inflammation are not only pathogenic factors, but also the result of m^6^A dysfunction. Elucidating the complex regulatory mechanism of m^6^A is helpful for the targeted treatment of bone related diseases. Nevertheless, these results enhance the understanding of the novel pathogenesis and phenotypes of osteoarthritis and provide new ideas for promoting personalized immunotherapy in the future.

## 5 Conclusion

This study reveals a potential regulatory mechanism of m^6^A methylation modification in the immune microenvironment of osteoarthritis. The diversity of m^6^A modification patterns is a factor contributing to the heterogeneity and complexity of the osteoarthritis immune microenvironment that cannot be ignored. Comprehensive analysis of m^6^A modification patterns in osteoarthritis allows us to gain a deeper understanding of the underlying mechanisms of osteoarthritis immune regulatory networks and guide more effective precision therapies.

## Data Availability

The original contributions presented in the study are included in the article/Supplementary Material, further inquiries can be directed to the corresponding authors.
