# Supplementary material for: N6-methyladenine regulator-mediated RNA methylation modification patterns in immune microenvironment regulation of osteoarthritis
Source: Front Genet. 2023 Jan 26;14:1113515. doi: 10.3389/fgene.2023.1113515 (PMC9908960; doi:10.3389/fgene.2023.1113515)
Supplement: Supplementary file 1 [file Table1.DOCX]

**Supplementary Figures and Tables**

**Supplementary Figures**

**
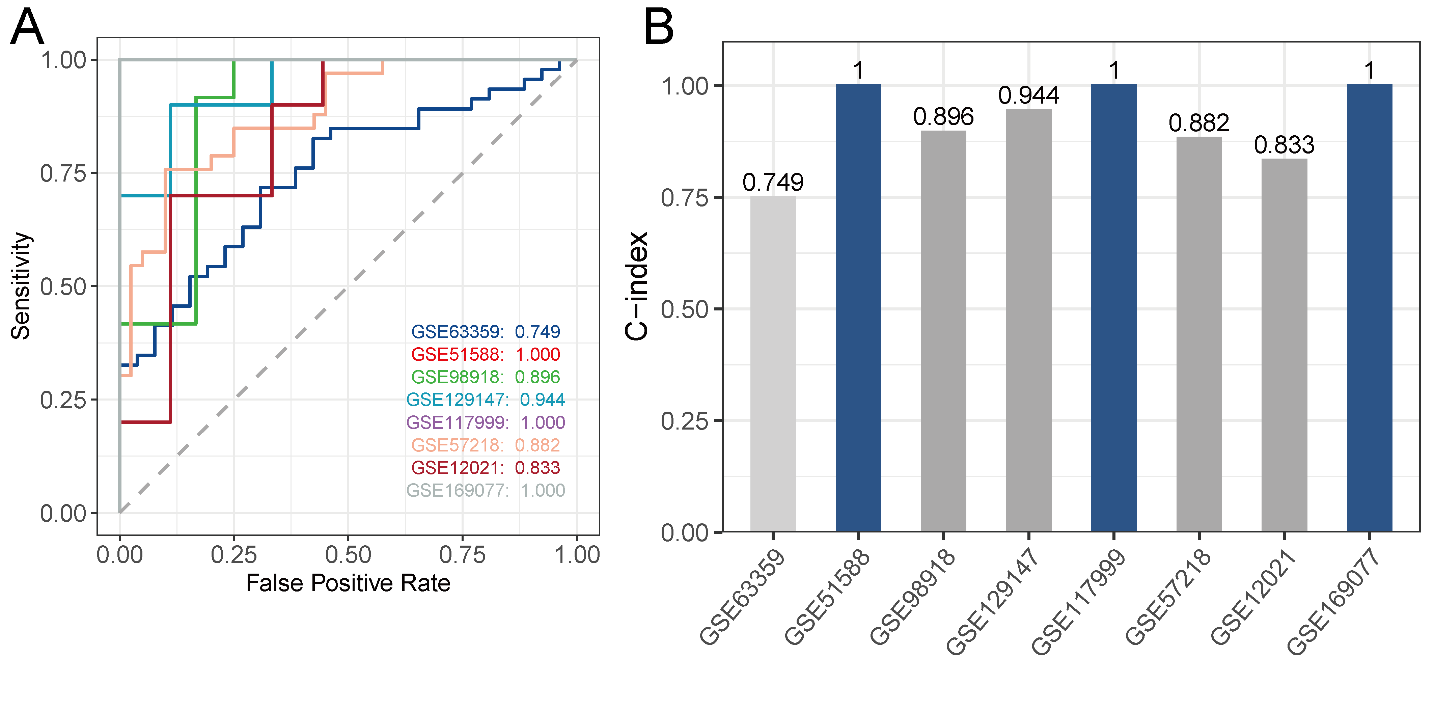
**Figure S1: ROC curves and C-indexes indicated that our model based on the expression of 6 m^6^A regulators had excellent diagnostic performance in external data sets.


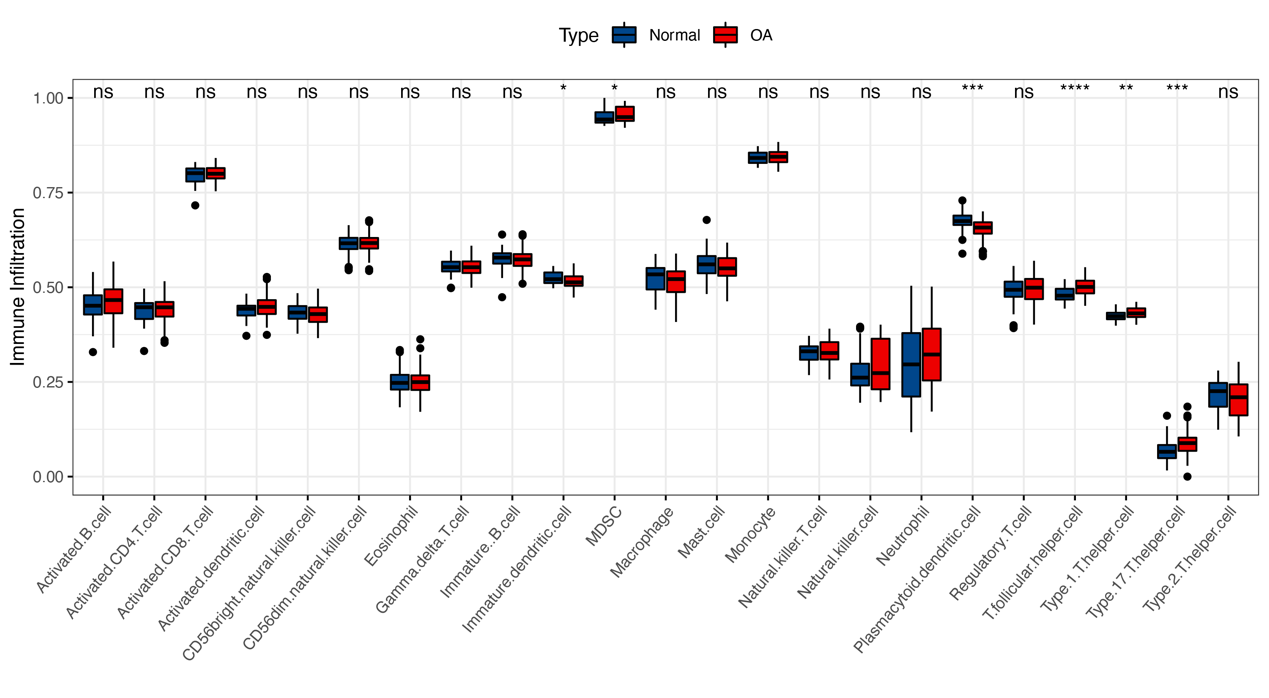


Figure S2: The differences in the abundance of each immune cell infiltration between healthy and osteoarthritis samples. Above * indicates *P* < 0.05, ** indicates *P* < 0.01, *** indicates *P* < 0.001, and ns indicates that the difference was not statistically significant.


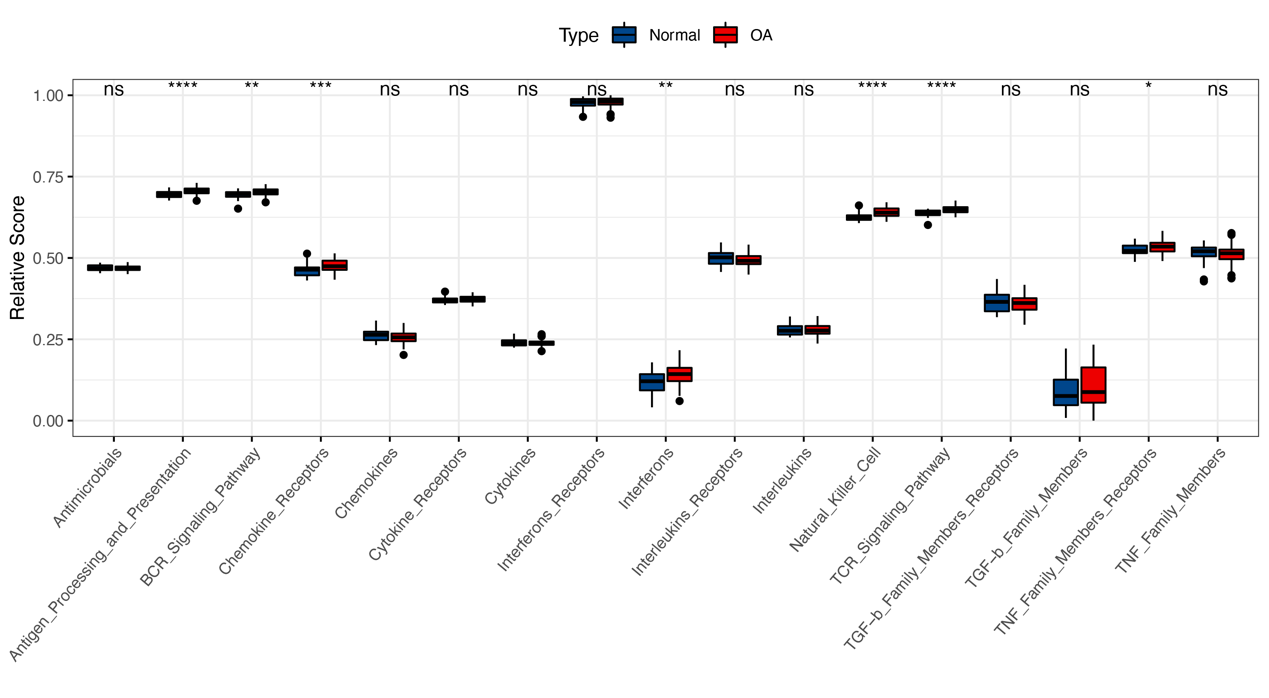


Figure S3: The activity differences of each immune response gene set between healthy and osteoarthritis samples. Above * indicates *P* < 0.05, ** indicates *P* < 0.01, *** indicates *P* < 0.001, and ns indicates that the difference was not statistically significant.


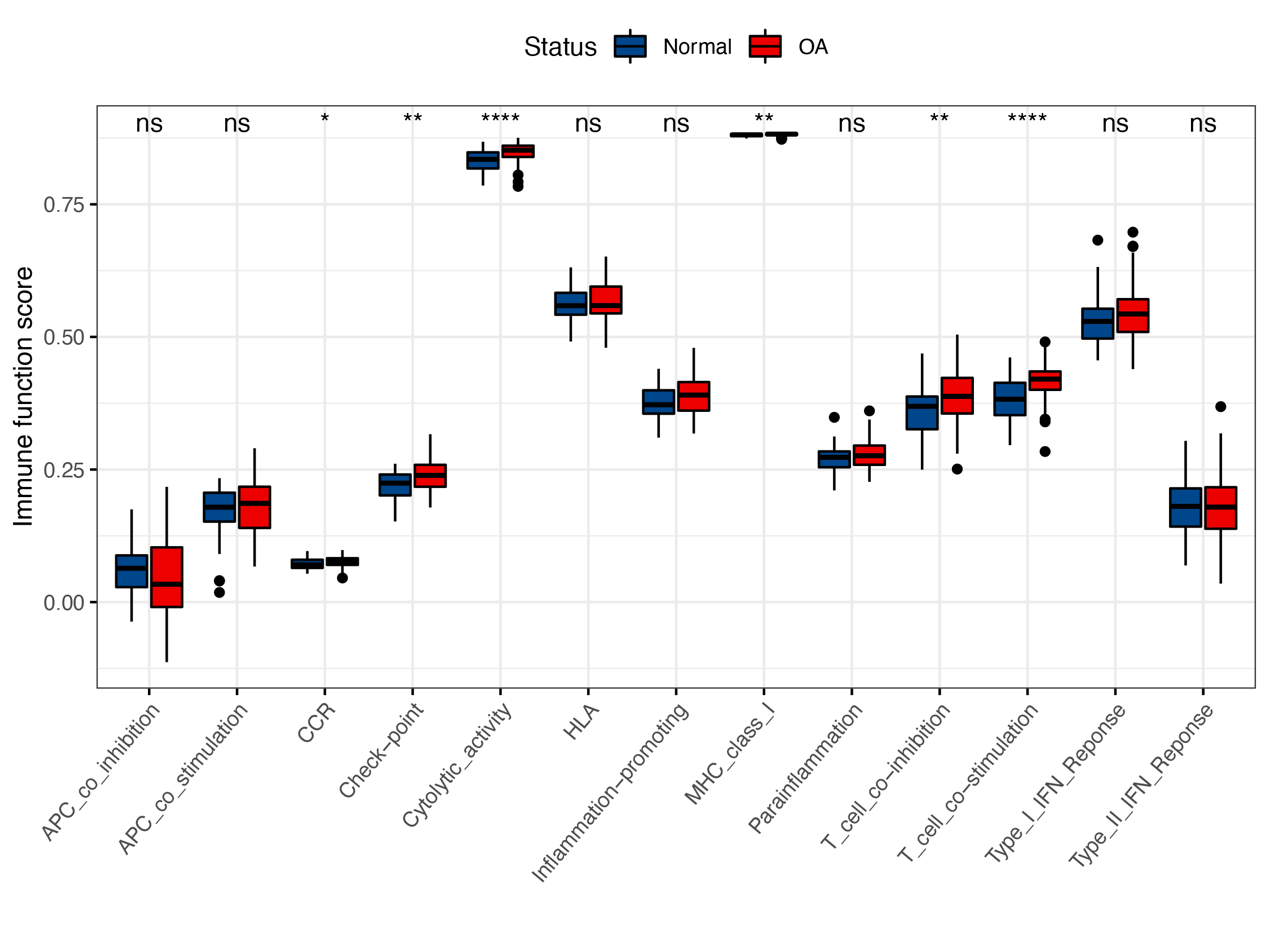


Figure S4: The expression differences of each immune function gene set between healthy and osteoarthritis samples. Above * indicates *P* < 0.05, ** indicates *P* < 0.01, *** indicates *P* < 0.001, and ns indicates that the difference was not statistically significant.

**Supplementary Tables**

Table S1 The age and gender information of the samples.

| ID | group | gender | age |
| --- | --- | --- | --- |
| GSM1182186 | OA | Female | 48 |
| GSM1182187 | OA | Female | 60 |
| GSM1182188 | OA | Female | 63 |
| GSM1182189 | OA | Female | 60 |
| GSM1182190 | OA | Female | 70 |
| GSM1182191 | OA | Female | 50 |
| GSM1182192 | OA | Female | 47 |
| GSM1182193 | OA | Female | 63 |
| GSM1182194 | OA | Female | 75 |
| GSM1182195 | OA | Female | 78 |
| GSM1182196 | OA | Female | 53 |
| GSM1182197 | OA | Female | 57 |
| GSM1182198 | OA | Female | 73 |
| GSM1182199 | OA | Female | 77 |
| GSM1182200 | OA | Female | 65 |
| GSM1182201 | OA | Female | 64 |
| GSM1182202 | OA | Female | 57 |
| GSM1182203 | OA | Female | 55 |
| GSM1182204 | OA | Female | 54 |
| GSM1182205 | OA | Female | 64 |
| GSM1182206 | OA | Female | 65 |
| GSM1182207 | OA | Female | 58 |
| GSM1182208 | OA | Female | 59 |
| GSM1182209 | OA | Female | 58 |
| GSM1182210 | OA | Female | 56 |
| GSM1182211 | OA | Female | 59 |
| GSM1182212 | OA | Female | 60 |
| GSM1182213 | OA | Female | 57 |
| GSM1182214 | OA | Female | 62 |
| GSM1182215 | OA | Female | 54 |
| GSM1182216 | OA | Female | 79 |
| GSM1182217 | OA | Female | 62 |
| GSM1182218 | OA | Female | 56 |
| GSM1182219 | OA | Female | 56 |
| GSM1182220 | OA | Female | 65 |
| GSM1182221 | OA | Female | 59 |
| GSM1182222 | OA | Female | 59 |
| GSM1182223 | OA | Female | 64 |
| GSM1182224 | OA | Female | 71 |
| GSM1182225 | OA | Female | 60 |
| GSM1182226 | OA | Female | 57 |
| GSM1182227 | OA | Female | 56 |
| GSM1182228 | OA | Female | 50 |
| GSM1182229 | OA | Female | 51 |
| GSM1182230 | OA | Female | 53 |
| GSM1182231 | OA | Female | 71 |
| GSM1182232 | OA | Female | 57 |
| GSM1182233 | OA | Female | 71 |
| GSM1182234 | OA | Female | 59 |
| GSM1182235 | OA | Female | 65 |
| GSM1182236 | OA | Female | 79 |
| GSM1182237 | OA | Female | 66 |
| GSM1182238 | OA | Female | 63 |
| GSM1182239 | OA | Female | 67 |
| GSM1182240 | OA | Female | 46 |
| GSM1182241 | OA | Female | 43 |
| GSM1182242 | OA | Female | 57 |
| GSM1182243 | OA | Female | 60 |
| GSM1182244 | OA | Female | 70 |
| GSM1182245 | OA | Female | 61 |
| GSM1182246 | OA | Female | 68 |
| GSM1182247 | OA | Female | 54 |
| GSM1182248 | OA | Female | 57 |
| GSM1182249 | OA | Female | 59 |
| GSM1182250 | OA | Female | 68 |
| GSM1182251 | OA | Female | 67 |
| GSM1182252 | OA | Female | 61 |
| GSM1182253 | OA | Female | 62 |
| GSM1182254 | OA | Female | 53 |
| GSM1182255 | OA | Female | 60 |
| GSM1182256 | OA | Female | 55 |
| GSM1182257 | OA | Female | 46 |
| GSM1182258 | OA | Female | 58 |
| GSM1182259 | OA | Female | 60 |
| GSM1182260 | OA | Female | 58 |
| GSM1182261 | OA | Female | 74 |
| GSM1182262 | OA | Female | 74 |
| GSM1182263 | OA | Female | 59 |
| GSM1182264 | OA | Female | 62 |
| GSM1182265 | OA | Female | 68 |
| GSM1182266 | OA | Female | 57 |
| GSM1182267 | OA | Female | 64 |
| GSM1182268 | OA | Female | 58 |
| GSM1182269 | OA | Female | 53 |
| GSM1182270 | OA | Female | 55 |
| GSM1182271 | OA | Female | 59 |
| GSM1182272 | OA | Female | 61 |
| GSM1182273 | OA | Female | 59 |
| GSM1182274 | OA | Male | 48 |
| GSM1182275 | OA | Female | 67 |
| GSM1182276 | OA | Female | 69 |
| GSM1182277 | OA | Female | 74 |
| GSM1182278 | OA | Female | 67 |
| GSM1182279 | OA | Female | 75 |
| GSM1182280 | OA | Female | 77 |
| GSM1182281 | OA | Female | 60 |
| GSM1182282 | OA | Female | 62 |
| GSM1182283 | OA | Female | 58 |
| GSM1182284 | OA | Female | 61 |
| GSM1182285 | OA | Female | 53 |
| GSM1182286 | OA | Female | 55 |
| GSM1182287 | OA | Female | 54 |
| GSM1182288 | OA | Female | 51 |
| GSM1182289 | OA | Female | 60 |
| GSM1182290 | OA | Female | 66 |
| GSM1182291 | OA | Female | 59 |
| GSM1182292 | Control | Female | 50 |
| GSM1182293 | Control | Female | 68 |
| GSM1182294 | Control | Female | 76 |
| GSM1182295 | Control | Female | 66 |
| GSM1182296 | Control | Female | 60 |
| GSM1182297 | Control | Male | 57 |
| GSM1182298 | Control | Female | 63 |
| GSM1182299 | Control | Female | 47 |
| GSM1182300 | Control | Female | 54 |
| GSM1182301 | Control | Female | 60 |
| GSM1182302 | Control | Male | 59 |
| GSM1182303 | Control | Female | 60 |
| GSM1182304 | Control | Female | 62 |
| GSM1182305 | Control | Female | 59 |
| GSM1182306 | Control | Female | 46 |
| GSM1182307 | Control | Female | 57 |
| GSM1182308 | Control | Male | 68 |
| GSM1182309 | Control | Female | 62 |
| GSM1182310 | Control | Male | 62 |
| GSM1182311 | Control | Male | 62 |
| GSM1182312 | Control | Female | 47 |
| GSM1182313 | Control | Male | 51 |
| GSM1182314 | Control | Female | 54 |
| GSM1182315 | Control | Male | 59 |
| GSM1182316 | Control | Female | 63 |
| GSM1182317 | Control | Male | 65 |
| GSM1182318 | Control | Female | 44 |
| GSM1182319 | Control | Female | 48 |
| GSM1182320 | Control | Female | 50 |
| GSM1182321 | Control | Female | 64 |
| GSM1182322 | Control | Female | 60 |
| GSM1182323 | Control | Male | 51 |
| GSM1182324 | Control | Female | 67 |

Table S2 The information for external data sets.

| ID | Group | Gender | Age | Tissue | Project |
| --- | --- | --- | --- | --- | --- |
| GSM1380887 | Control | Male | 16 | Cartilage | GSE57218 |
| GSM1380888 | Control | Female | 18 | Cartilage | GSE57218 |
| GSM1380890 | Control | Female | 78 | Cartilage | GSE57218 |
| GSM1380892 | Control | Male | 56 | Cartilage | GSE57218 |
| GSM1380894 | Control | Male | 55 | Cartilage | GSE57218 |
| GSM1380896 | Control | Female | 61 | Cartilage | GSE57218 |
| GSM1380898 | Control | Female | 79 | Cartilage | GSE57218 |
| GSM1380900 | Control | Female | 80 | Cartilage | GSE57218 |
| GSM1380902 | Control | Male | 67 | Cartilage | GSE57218 |
| GSM1380904 | Control | Female | 70 | Cartilage | GSE57218 |
| GSM1380906 | Control | Female | 77 | Cartilage | GSE57218 |
| GSM1380908 | Control | Female | 58 | Cartilage | GSE57218 |
| GSM1380910 | Control | Female | 74 | Cartilage | GSE57218 |
| GSM1380912 | Control | Male | 73 | Cartilage | GSE57218 |
| GSM1380914 | Control | Male | 63 | Cartilage | GSE57218 |
| GSM1380916 | Control | Female | 76 | Cartilage | GSE57218 |
| GSM1380917 | Control | Female | 64 | Cartilage | GSE57218 |
| GSM1380919 | Control | Male | 54 | Cartilage | GSE57218 |
| GSM1380921 | Control | Male | 58 | Cartilage | GSE57218 |
| GSM1380923 | Control | Female | 64 | Cartilage | GSE57218 |
| GSM1380925 | Control | Female | 75 | Cartilage | GSE57218 |
| GSM1380927 | Control | Female | 67 | Cartilage | GSE57218 |
| GSM1380929 | Control | Male | 71 | Cartilage | GSE57218 |
| GSM1380931 | Control | Female | 59 | Cartilage | GSE57218 |
| GSM1380933 | Control | Female | 55 | Cartilage | GSE57218 |
| GSM1380935 | Control | Male | 74 | Cartilage | GSE57218 |
| GSM1380937 | Control | Male | 55 | Cartilage | GSE57218 |
| GSM1380939 | Control | Female | 54 | Cartilage | GSE57218 |
| GSM1380941 | Control | Male | 65 | Cartilage | GSE57218 |
| GSM1380943 | Control | Female | 66 | Cartilage | GSE57218 |
| GSM1380945 | Control | Female | 69 | Cartilage | GSE57218 |
| GSM1380947 | Control | Male | 70 | Cartilage | GSE57218 |
| GSM1380949 | Control | Male | 62 | Cartilage | GSE57218 |
| GSM1380951 | Control | Female | 68 | Cartilage | GSE57218 |
| GSM1380953 | Control | Female | 56 | Cartilage | GSE57218 |
| GSM1380955 | Control | Female | 75 | Cartilage | GSE57218 |
| GSM1380956 | Control | Female | 80 | Cartilage | GSE57218 |
| GSM1380957 | Control | Female | 76 | Cartilage | GSE57218 |
| GSM1380958 | Control | Female | 15 | Cartilage | GSE57218 |
| GSM1380959 | Control | Female | 16 | Cartilage | GSE57218 |
| GSM1380889 | OA | Female | 78 | Cartilage | GSE57218 |
| GSM1380891 | OA | Male | 56 | Cartilage | GSE57218 |
| GSM1380893 | OA | Male | 55 | Cartilage | GSE57218 |
| GSM1380895 | OA | Female | 61 | Cartilage | GSE57218 |
| GSM1380897 | OA | Female | 79 | Cartilage | GSE57218 |
| GSM1380899 | OA | Female | 80 | Cartilage | GSE57218 |
| GSM1380901 | OA | Male | 67 | Cartilage | GSE57218 |
| GSM1380903 | OA | Female | 70 | Cartilage | GSE57218 |
| GSM1380905 | OA | Female | 77 | Cartilage | GSE57218 |
| GSM1380907 | OA | Female | 58 | Cartilage | GSE57218 |
| GSM1380909 | OA | Female | 74 | Cartilage | GSE57218 |
| GSM1380911 | OA | Male | 73 | Cartilage | GSE57218 |
| GSM1380913 | OA | Male | 63 | Cartilage | GSE57218 |
| GSM1380915 | OA | Female | 76 | Cartilage | GSE57218 |
| GSM1380918 | OA | Male | 54 | Cartilage | GSE57218 |
| GSM1380920 | OA | Male | 58 | Cartilage | GSE57218 |
| GSM1380922 | OA | Female | 64 | Cartilage | GSE57218 |
| GSM1380924 | OA | Female | 75 | Cartilage | GSE57218 |
| GSM1380926 | OA | Female | 67 | Cartilage | GSE57218 |
| GSM1380928 | OA | Male | 71 | Cartilage | GSE57218 |
| GSM1380930 | OA | Female | 59 | Cartilage | GSE57218 |
| GSM1380932 | OA | Female | 55 | Cartilage | GSE57218 |
| GSM1380934 | OA | Male | 74 | Cartilage | GSE57218 |
| GSM1380936 | OA | Male | 55 | Cartilage | GSE57218 |
| GSM1380938 | OA | Female | 54 | Cartilage | GSE57218 |
| GSM1380940 | OA | Male | 65 | Cartilage | GSE57218 |
| GSM1380942 | OA | Female | 66 | Cartilage | GSE57218 |
| GSM1380944 | OA | Female | 69 | Cartilage | GSE57218 |
| GSM1380946 | OA | Male | 70 | Cartilage | GSE57218 |
| GSM1380948 | OA | Male | 62 | Cartilage | GSE57218 |
| GSM1380950 | OA | Female | 68 | Cartilage | GSE57218 |
| GSM1380952 | OA | Female | 56 | Cartilage | GSE57218 |
| GSM1380954 | OA | Female | 75 | Cartilage | GSE57218 |
| GSM3316962 | Control | Female | 37 | Cartilage | GSE117999 |
| GSM3316963 | Control | Male | 45 | Cartilage | GSE117999 |
| GSM3316964 | Control | Female | 62 | Cartilage | GSE117999 |
| GSM3316965 | Control | Female | 58 | Cartilage | GSE117999 |
| GSM3316966 | Control | Female | 31 | Cartilage | GSE117999 |
| GSM3316967 | Control | Male | 53 | Cartilage | GSE117999 |
| GSM3316968 | Control | Male | 50 | Cartilage | GSE117999 |
| GSM3316969 | Control | Female | 65 | Cartilage | GSE117999 |
| GSM3316971 | Control | Male | 43 | Cartilage | GSE117999 |
| GSM3316973 | Control | Male | 53 | Cartilage | GSE117999 |
| GSM3316974 | OA | Male | 57 | Cartilage | GSE117999 |
| GSM3316975 | OA | Female | 64 | Cartilage | GSE117999 |
| GSM3316977 | OA | Female | 62 | Cartilage | GSE117999 |
| GSM3316978 | OA | Male | 80 | Cartilage | GSE117999 |
| GSM3316979 | OA | Female | 67 | Cartilage | GSE117999 |
| GSM3316981 | OA | Female | 64 | Cartilage | GSE117999 |
| GSM3316982 | OA | Male | 61 | Cartilage | GSE117999 |
| GSM3316983 | OA | Female | 62 | Cartilage | GSE117999 |
| GSM3316984 | OA | Female | 79 | Cartilage | GSE117999 |
| GSM3316985 | OA | Female | 64 | Cartilage | GSE117999 |
| GSM302859 | Control | Male | 61 | Synovial | GSE12021 |
| GSM302864 | Control | Male | 64 | Synovial | GSE12021 |
| GSM302866 | Control | Female | 88 | Synovial | GSE12021 |
| GSM302870 | Control | Male | 65 | Synovial | GSE12021 |
| GSM302876 | OA | Female | 77 | Synovial | GSE12021 |
| GSM302880 | OA | Female | 71 | Synovial | GSE12021 |
| GSM302930 | OA | Female | 76 | Synovial | GSE12021 |
| GSM303326 | OA | Female | 61 | Synovial | GSE12021 |
| GSM303341 | OA | Female | 75 | Synovial | GSE12021 |
| GSM303356 | OA | Male | 78 | Synovial | GSE12021 |
| GSM303358 | OA | Male | 64 | Synovial | GSE12021 |
| GSM303360 | OA | Female | 71 | Synovial | GSE12021 |
| GSM303362 | OA | Female | 80 | Synovial | GSE12021 |
| GSM303370 | OA | Female | 66 | Synovial | GSE12021 |
| GSM303522 | Control | Male | 53 | Synovial | GSE12021 |
| GSM303523 | Control | Female | 29 | Synovial | GSE12021 |
| GSM303525 | Control | Male | 17 | Synovial | GSE12021 |
| GSM303531 | Control | Male | 39 | Synovial | GSE12021 |
| GSM303533 | Control | Male | 36 | Synovial | GSE12021 |
| GSM5176138 | Control | NA | NA | Cartilage | GSE169077 |
| GSM5176139 | Control | NA | NA | Cartilage | GSE169077 |
| GSM5176140 | Control | NA | NA | Cartilage | GSE169077 |
| GSM5176141 | Control | NA | NA | Cartilage | GSE169077 |
| GSM5176142 | Control | NA | NA | Cartilage | GSE169077 |
| GSM5176143 | OA | NA | NA | Cartilage | GSE169077 |
| GSM5176144 | OA | NA | NA | Cartilage | GSE169077 |
| GSM5176145 | OA | NA | NA | Cartilage | GSE169077 |
| GSM5176146 | OA | NA | NA | Cartilage | GSE169077 |
| GSM5176147 | OA | NA | NA | Cartilage | GSE169077 |
| GSM5176148 | OA | NA | NA | Cartilage | GSE169077 |
| GSM2627518 | Control | Female | 37 | Cartilage | GSE98918 |
| GSM2627519 | Control | Male | 45 | Cartilage | GSE98918 |
| GSM2627520 | Control | Female | 62 | Cartilage | GSE98918 |
| GSM2627521 | Control | Female | 58 | Cartilage | GSE98918 |
| GSM2627522 | Control | Female | 31 | Cartilage | GSE98918 |
| GSM2627523 | Control | Male | 53 | Cartilage | GSE98918 |
| GSM2627524 | Control | Male | 50 | Cartilage | GSE98918 |
| GSM2627525 | Control | Female | 65 | Cartilage | GSE98918 |
| GSM2627526 | Control | Male | 53 | Cartilage | GSE98918 |
| GSM2627527 | Control | Male | 43 | Cartilage | GSE98918 |
| GSM2627528 | Control | Male | 40 | Cartilage | GSE98918 |
| GSM2627529 | Control | Male | 53 | Cartilage | GSE98918 |
| GSM2627530 | OA | Male | 57 | Cartilage | GSE98918 |
| GSM2627531 | OA | Female | 64 | Cartilage | GSE98918 |
| GSM2627532 | OA | Female | 53 | Cartilage | GSE98918 |
| GSM2627533 | OA | Female | 62 | Cartilage | GSE98918 |
| GSM2627534 | OA | Male | 80 | Cartilage | GSE98918 |
| GSM2627535 | OA | Female | 67 | Cartilage | GSE98918 |
| GSM2627536 | OA | Female | 70 | Cartilage | GSE98918 |
| GSM2627537 | OA | Female | 64 | Cartilage | GSE98918 |
| GSM2627538 | OA | Male | 61 | Cartilage | GSE98918 |
| GSM2627539 | OA | Female | 62 | Cartilage | GSE98918 |
| GSM2627540 | OA | Female | 79 | Cartilage | GSE98918 |
| GSM2627541 | OA | Female | 64 | Cartilage | GSE98918 |
| GSM1248759 | Control | Female | 35 | Subchondral Bone | GSE51588 |
| GSM1248760 | Control | Female | 35 | Subchondral Bone | GSE51588 |
| GSM1248761 | Control | Male | 41 | Subchondral Bone | GSE51588 |
| GSM1248762 | Control | Female | 22 | Subchondral Bone | GSE51588 |
| GSM1248763 | Control | Male | 59 | Subchondral Bone | GSE51588 |
| GSM1248764 | Control | Female | 35 | Subchondral Bone | GSE51588 |
| GSM1248765 | Control | Female | 35 | Subchondral Bone | GSE51588 |
| GSM1248766 | Control | Male | 41 | Subchondral Bone | GSE51588 |
| GSM1248767 | Control | Female | 22 | Subchondral Bone | GSE51588 |
| GSM1248768 | Control | Male | 59 | Subchondral Bone | GSE51588 |
| GSM1248769 | OA | Female | 55 | Subchondral Bone | GSE51588 |
| GSM1248770 | OA | Male | 68 | Subchondral Bone | GSE51588 |
| GSM1248771 | OA | Male | 66 | Subchondral Bone | GSE51588 |
| GSM1248772 | OA | Female | 71 | Subchondral Bone | GSE51588 |
| GSM1248773 | OA | Female | 71 | Subchondral Bone | GSE51588 |
| GSM1248774 | OA | Male | 81 | Subchondral Bone | GSE51588 |
| GSM1248775 | OA | Female | 67 | Subchondral Bone | GSE51588 |
| GSM1248776 | OA | Female | 59 | Subchondral Bone | GSE51588 |
| GSM1248777 | OA | Male | 77 | Subchondral Bone | GSE51588 |
| GSM1248778 | OA | Female | 71 | Subchondral Bone | GSE51588 |
| GSM1248779 | OA | Female | 63 | Subchondral Bone | GSE51588 |
| GSM1248780 | OA | Female | 53 | Subchondral Bone | GSE51588 |
| GSM1248781 | OA | Female | 77 | Subchondral Bone | GSE51588 |
| GSM1248782 | OA | Male | 68 | Subchondral Bone | GSE51588 |
| GSM1248783 | OA | Male | 75 | Subchondral Bone | GSE51588 |
| GSM1248784 | OA | Female | 67 | Subchondral Bone | GSE51588 |
| GSM1248785 | OA | Male | 89 | Subchondral Bone | GSE51588 |
| GSM1248786 | OA | Female | 68 | Subchondral Bone | GSE51588 |
| GSM1248787 | OA | Female | 76 | Subchondral Bone | GSE51588 |
| GSM1248788 | OA | Male | 81 | Subchondral Bone | GSE51588 |
| GSM1248789 | OA | Female | 55 | Subchondral Bone | GSE51588 |
| GSM1248790 | OA | Male | 68 | Subchondral Bone | GSE51588 |
| GSM1248791 | OA | Male | 66 | Subchondral Bone | GSE51588 |
| GSM1248792 | OA | Male | 68 | Subchondral Bone | GSE51588 |
| GSM1248793 | OA | Female | 63 | Subchondral Bone | GSE51588 |
| GSM1248794 | OA | Male | 65 | Subchondral Bone | GSE51588 |
| GSM1248795 | OA | Female | 71 | Subchondral Bone | GSE51588 |
| GSM1248796 | OA | Female | 60 | Subchondral Bone | GSE51588 |
| GSM1248797 | OA | Female | 63 | Subchondral Bone | GSE51588 |
| GSM1248798 | OA | Female | 83 | Subchondral Bone | GSE51588 |
| GSM1248799 | OA | Female | 77 | Subchondral Bone | GSE51588 |
| GSM1248800 | OA | Male | 52 | Subchondral Bone | GSE51588 |
| GSM1248801 | OA | Male | 74 | Subchondral Bone | GSE51588 |
| GSM1248802 | OA | Male | 68 | Subchondral Bone | GSE51588 |
| GSM1248803 | OA | Female | 68 | Subchondral Bone | GSE51588 |
| GSM1248804 | OA | Male | 69 | Subchondral Bone | GSE51588 |
| GSM1248805 | OA | Male | 89 | Subchondral Bone | GSE51588 |
| GSM1248806 | OA | Female | 68 | Subchondral Bone | GSE51588 |
| GSM1248807 | OA | Female | 76 | Subchondral Bone | GSE51588 |
| GSM1248808 | OA | Male | 81 | Subchondral Bone | GSE51588 |
| GSM1546587 | Control | Female | 46 | PBMC | GSE63359 |
| GSM1546588 | Control | Female | 54 | PBMC | GSE63359 |
| GSM1546589 | Control | Female | 54 | PBMC | GSE63359 |
| GSM1546590 | Control | Male | 56 | PBMC | GSE63359 |
| GSM1546591 | Control | Female | 54 | PBMC | GSE63359 |
| GSM1546592 | Control | Male | 52 | PBMC | GSE63359 |
| GSM1546593 | Control | Female | 62 | PBMC | GSE63359 |
| GSM1546594 | Control | Female | 49 | PBMC | GSE63359 |
| GSM1546595 | Control | Female | 55 | PBMC | GSE63359 |
| GSM1546596 | Control | Female | 49 | PBMC | GSE63359 |
| GSM1546597 | Control | Female | 56 | PBMC | GSE63359 |
| GSM1546598 | Control | Male | 57 | PBMC | GSE63359 |
| GSM1546599 | Control | Female | 49 | PBMC | GSE63359 |
| GSM1546600 | Control | Female | 58 | PBMC | GSE63359 |
| GSM1546601 | Control | Female | 66 | PBMC | GSE63359 |
| GSM1546602 | Control | Female | 31 | PBMC | GSE63359 |
| GSM1546603 | Control | Female | 58 | PBMC | GSE63359 |
| GSM1546604 | Control | Male | 48 | PBMC | GSE63359 |
| GSM1546605 | Control | Male | 65 | PBMC | GSE63359 |
| GSM1546606 | Control | Female | 50 | PBMC | GSE63359 |
| GSM1546607 | Control | Male | 52 | PBMC | GSE63359 |
| GSM1546608 | Control | Female | 87 | PBMC | GSE63359 |
| GSM1546609 | Control | Male | 50 | PBMC | GSE63359 |
| GSM1546610 | Control | Female | 49 | PBMC | GSE63359 |
| GSM1546611 | Control | Female | 53 | PBMC | GSE63359 |
| GSM1546612 | Control | Female | 59 | PBMC | GSE63359 |
| GSM1546613 | OA | Female | 69 | PBMC | GSE63359 |
| GSM1546614 | OA | Male | 74 | PBMC | GSE63359 |
| GSM1546615 | OA | Female | 53 | PBMC | GSE63359 |
| GSM1546616 | OA | Female | 64 | PBMC | GSE63359 |
| GSM1546617 | OA | Female | 74 | PBMC | GSE63359 |
| GSM1546618 | OA | Female | 52 | PBMC | GSE63359 |
| GSM1546619 | OA | Female | 70 | PBMC | GSE63359 |
| GSM1546620 | OA | Male | 80 | PBMC | GSE63359 |
| GSM1546621 | OA | Female | 83 | PBMC | GSE63359 |
| GSM1546622 | OA | Male | 77 | PBMC | GSE63359 |
| GSM1546623 | OA | Female | 49 | PBMC | GSE63359 |
| GSM1546624 | OA | Female | 61 | PBMC | GSE63359 |
| GSM1546625 | OA | Female | 51 | PBMC | GSE63359 |
| GSM1546626 | OA | Female | 75 | PBMC | GSE63359 |
| GSM1546627 | OA | Male | 72 | PBMC | GSE63359 |
| GSM1546628 | OA | Female | 84 | PBMC | GSE63359 |
| GSM1546629 | OA | Female | 72 | PBMC | GSE63359 |
| GSM1546630 | OA | Female | 66 | PBMC | GSE63359 |
| GSM1546631 | OA | Female | 64 | PBMC | GSE63359 |
| GSM1546632 | OA | Male | 56 | PBMC | GSE63359 |
| GSM1546633 | OA | Female | 75 | PBMC | GSE63359 |
| GSM1546634 | OA | Female | 56 | PBMC | GSE63359 |
| GSM1546635 | OA | Female | 79 | PBMC | GSE63359 |
| GSM1546636 | OA | Male | 72 | PBMC | GSE63359 |
| GSM1546637 | OA | Male | 56 | PBMC | GSE63359 |
| GSM1546638 | OA | Female | 65 | PBMC | GSE63359 |
| GSM1546639 | OA | Female | 53 | PBMC | GSE63359 |
| GSM1546640 | OA | Female | 47 | PBMC | GSE63359 |
| GSM1546641 | OA | Male | 57 | PBMC | GSE63359 |
| GSM1546642 | OA | Male | 50 | PBMC | GSE63359 |
| GSM1546643 | OA | Female | 50 | PBMC | GSE63359 |
| GSM1546644 | OA | Female | 78 | PBMC | GSE63359 |
| GSM1546645 | OA | Female | 76 | PBMC | GSE63359 |
| GSM1546646 | OA | Female | 52 | PBMC | GSE63359 |
| GSM1546647 | OA | Male | 80 | PBMC | GSE63359 |
| GSM1546648 | OA | Female | 56 | PBMC | GSE63359 |
| GSM1546649 | OA | Female | 68 | PBMC | GSE63359 |
| GSM1546650 | OA | Male | 62 | PBMC | GSE63359 |
| GSM1546651 | OA | Female | 78 | PBMC | GSE63359 |
| GSM1546652 | OA | Female | 73 | PBMC | GSE63359 |
| GSM1546653 | OA | Female | 62 | PBMC | GSE63359 |
| GSM1546654 | OA | Male | 57 | PBMC | GSE63359 |
| GSM1546655 | OA | Male | 74 | PBMC | GSE63359 |
| GSM1546656 | OA | Male | 74 | PBMC | GSE63359 |
| GSM1546657 | OA | Female | 69 | PBMC | GSE63359 |
| GSM1546658 | OA | Female | 54 | PBMC | GSE63359 |
| GSM3701266 | OA | NA | NA | Cartilage | GSE129147 |
| GSM3701267 | OA | NA | NA | Cartilage | GSE129147 |
| GSM3701268 | OA | NA | NA | Cartilage | GSE129147 |
| GSM3701269 | OA | NA | NA | Cartilage | GSE129147 |
| GSM3701270 | OA | NA | NA | Cartilage | GSE129147 |
| GSM3701271 | OA | NA | NA | Cartilage | GSE129147 |
| GSM3701272 | OA | NA | NA | Cartilage | GSE129147 |
| GSM3701273 | OA | NA | NA | Cartilage | GSE129147 |
| GSM3701274 | OA | NA | NA | Cartilage | GSE129147 |
| GSM3701275 | OA | NA | NA | Cartilage | GSE129147 |
| GSM3701276 | Control | NA | NA | Cartilage | GSE129147 |
| GSM3701277 | Control | NA | NA | Cartilage | GSE129147 |
| GSM3701278 | Control | NA | NA | Cartilage | GSE129147 |
| GSM3701279 | Control | NA | NA | Cartilage | GSE129147 |
| GSM3701280 | Control | NA | NA | Cartilage | GSE129147 |
| GSM3701281 | Control | NA | NA | Cartilage | GSE129147 |
| GSM3701282 | Control | NA | NA | Cartilage | GSE129147 |
| GSM3701283 | Control | NA | NA | Cartilage | GSE129147 |
| GSM3701284 | Control | NA | NA | Cartilage | GSE129147 |

Table S3 The univariate regression results of 23 m^6^A regulators.

| Genes | OR(95%CI) | *P*-value |
| --- | --- | --- |
| METTL3 | 0.08(0.01-0.5) | 0.01 |
| METTL14 | 0.63(0.02-26.1) | 0.81 |
| METTL16 | 0.57(0.13-2.6) | 0.47 |
| WTAP | 0.34(0.08-1.38) | 0.13 |
| VIRMA | 1.78(0.5-6.34) | 0.38 |
| ZC3H13 | 1.01(0.01-76.73) | 1 |
| RBM15 | 7.71(1.52-39.2) | 0.01 |
| RBM15B | 2.94(1.03-8.37) | 0.04 |
| YTHDC1 | 1.15(0.41-3.24) | 0.79 |
| YTHDC2 | 0.21(0.03-1.58) | 0.13 |
| YTHDF1 | 4.44(0.68-28.9) | 0.12 |
| YTHDF2 | 3.85(0.7-21.05) | 0.12 |
| YTHDF3 | 0.44(0.06-3.12) | 0.41 |
| HNRNPC | 0.15(0.02-0.91) | 0.04 |
| FMR1 | 4.91(0.01-2443.26) | 0.62 |
| LRPPRC | 0.64(0.23-1.81) | 0.4 |
| HNRNPA2B1 | 0.66(0.23-1.96) | 0.46 |
| IGFBP1 | 2275.74(6.62-782600.05) | 0.01 |
| IGFBP2 | 0.18(0.04-0.86) | 0.03 |
| IGFBP3 | 46.5(4.36-496.37) | <0.001 |
| RBMX | 1.9(0.24-15.35) | 0.55 |
| FTO | 4.68(0.68-32.2) | 0.12 |
| ALKBH5 | 1.08(0.12-9.93) | 0.94 |

Table S4 The multivariate regression results of 12 m^6^A regulators.

| Genes | OR(95%CI) | *P*-value |
| --- | --- | --- |
| METTL3 | -3.22(-5.99~-0.7) | 0.016 |
| RBM15 | 4.14(1.89~6.69) | 0.001 |
| HNRNPC | -2.65(-5.31~-0.3) | 0.036 |
| IGFBP1 | 12.12(3.55~21.67) | 0.008 |
| FTO | 8.14(4.66~12.3) | <0.001 |
| IGFBP2 | -3.56(-5.94~-1.46) | 0.002 |

Table S5 The m^6^A phenotype-related immune genes.

| Genes | Genes | Genes |
| --- | --- | --- |
| PTPN11 | JAK2 | TNFRSF14 |
| TFRC | CREB1 | RORA |
| PIK3R1 | IL12RB1 | SEM1 |
| IL17RD | ERAP2 | NOD1 |
| PSMD4 | ISG20L2 | GMFG |
| IL6ST | FYN | PML |
| PSMC6 | AP3B1 | ZC3HAV1 |
| KRAS | IFITM1 | TAP1 |
| TXK | CANX | STAT1 |
| RABEP1 | DAXX | CD1A |
| TANK | ICAM2 | CYBB |
| CHUK | CMTM6 | PTGER4 |
| TNFRSF1A | SEMA4B | TK2 |
| PIK3R2 | IKBKE | NLRX1 |
| CYLD | ACVR1B | PSMD5 |
| AKT1 | ICOS | BMPR2 |
| CYSLTR1 | CETP | MANF |
| IREB2 | PSMD6 | PSMD1 |
| PSMB8 | NFAT5 | TAP2 |

Table S6 Distinct modification patterns matched their related genes and were divided into five gene modules with different colors.

| Module Color | Blue | Brown | Grey | Turquoise | Yellow |
| --- | --- | --- | --- | --- | --- |
| Genes | ETS1 | GNAI2 | TIMP1 | UBB | CREB1 |
|  | EIF3G | AP1S2 | LAMP1 | COX4I1 | SRSF2 |
|  | SEM1 | SEPTIN2 | ARF1 | TSPO | ERAP2 |
|  | PHF11 | HSP90B1 | SNU13 | EEF1B2 | TNFRSF1A |
|  | RBM5 | PTBP3 | CDKN1A | CCNI | ARGLU1 |
|  | FNBP4 | DARS1 | CLDND1 | SNRPD2 | CATSPER2 |
|  | PTPRE | TACC1 | ALYREF | NUCB1 | URI1 |
|  | STX16 | ARHGEF6 | MOB3C | AP2S1 | EIF4A3 |
|  | STAT1 | PRKAR1A | GPS2 | JTB | DDX3X |
|  | VEZF1 | HNRNPR | COG2 | IFITM1 | NBPF11 |
|  | BCL11B | TUG1 | CUL1 | ATP5MG | COA3 |
|  | CANX | PPP2R1A | CYTIP | SLC25A3 | LRRFIP1 |
|  | DENND11 | SYF2 | FRG1 | RPL29 | CPNE3 |
|  | TRIM44 | RBL2 | TATDN1 | LASP1 | ZNF581 |
|  | CDC16 | LSM14A | C8orf34 | GLIPR2 | SNRPA1 |
|  | SCARB2 | ZC3HAV1 | BEGAIN | ICAM3 | THOC7 |
|  | BCL2 | CMTM6 |  | HK1 | FAM89B |
|  | SUPT16H | MMADHC | | VAMP8 | PNRC2 |
|  | PATL1 | NUCKS1 |  | FLOT2 | PPA2 |
|  | SPEN | AP3B1 |  | BTG1 | BCKDHA |
|  | DYNC1LI2 | GATD3A |  | TNFRSF14 | FYB1 |
|  | HERC1 | DDX18 |  | DNAJC8 | TTC3 |
|  | CKAP5 | RBMS1 |  | STK24 | SSR1 |
|  | VPS41 | UBE2M |  | EZR | UGP2 |
|  | CCDC90B | ASAP1 |  | GIMAP7 | DOCK10 |
|  | RNF38 | RALA |  | YWHAB | FAM117B |
|  | C18orf25 | PUM2 |  | TAP1 | NAP1L1 |
|  | SYPL1 | CRBN |  | SSU72 | GCA |
|  | ARID5B | EFR3A |  | FAM177A1 | GRIPAP1 |
|  | USP24 | CAPN1 |  | ICAM2 | RRAS |
|  | FAM217B | HECTD1 |  | RPN1 | YRDC |
|  | FOXJ3 | PYHIN1 |  | SH3KBP1 | MFSD5 |
|  | CTNNBL1 | PDK3 |  | VPS51 | CDC40 |
|  | RBM33 | PRRC2C |  | RNF7 | TOMM70 |
|  | AP3M1 | PHF3 |  | EIF3B | DDX21 |
|  | ZNF512 | ASNSD1 |  | BANF1 | ZBTB33 |
|  | OSBPL9 | SETD2 |  | CS | UPF2 |
|  | BMS1 | RAB3GAP1 | | BANP | METAP2 |
|  | ZNF330 | GGNBP2 |  | ATP6V1E1 | GNA13 |
|  | PSMD1 | SAMD3 |  | HNRNPAB | BLZF1 |
|  | GALC | KLF9 |  | PSMB8 | SHKBP1 |
|  | PSTPIP1 | PPP4R3A |  | PPP1CA | PAFAH1B1 |
|  | KDELR1 | ZC3H7A |  | GABARAPL2 | WDFY1 |
|  | AGAP6 | EBAG9 |  | ECH1 | VAMP7 |
|  | C1orf131 | TDG |  | ARHGAP30 | DOCK11 |
|  | BBS2 | PSMC6 |  | CYTH4 | MAGT1 |
|  | VAMP1 | WASHC5 |  | SNRPB | STYX |
|  | LYRM2 | NUS1 |  | PSMB6 | VBP1 |
|  | TRIB2 | RNMT |  | PUF60 | PPP1CB |
|  | SLBP | BNIP3L |  | RTF2 | XPO1 |
|  | GRSF1 | PCMTD2 |  | COMT | VPS26A |
|  | BLCAP | RAB5C |  | NECAP2 | PSME4 |
|  | KLHL12 | LRBA |  | EIF4H | KIDINS220 |
|  | LEMD3 | FBXO11 |  | CALM2 | ASXL2 |
|  | BTBD6 | AK3 |  | JMJD8 | TRA2B |
|  | VPS45 | BCL11A |  | BRPF1 | APAF1 |
|  | AP1G2 | MRPS31 |  | NDUFA11 | AOC4P |
|  | CARD8 | ANGEL2 |  | PSMD4 | NAE1 |
|  | FBXO33 | LBHD1 |  | TMEM205 | IVNS1ABP |
|  | NUP160 | CDK19 |  | ZNHIT3 | PAPOLA |
|  | BRIX1 | TMEM167B | | BRMS1 | TANK |
|  | OARD1 | RBM42 |  | NDUFS7 | FKBP14 |
|  | HSPB11 | AKTIP |  | FAM50A | LAMTOR3 |
|  | TAF4 | FUBP3 |  | KHDRBS1 | BBX |
|  | METTL3 | PPP6R3 |  | NOL12 | TFRC |
|  | RAB27A | SERBP1 |  | HSBP1 | CERT1 |
|  | ZFAND6 | WWP1 |  | COLGALT1 | USP9X |
|  | FAM172A | MRPL53 |  | UBE2F | EML4 |
|  | SECISBP2 | PCNX4 |  | APRT | FOXN2 |
|  | FXR1 | ZYG11B |  | AKR1A1 | SLC30A7 |
|  | MAPK9 | HNRNPDL | | TINF2 | PIK3R1 |
|  | TFAM | GNL3 |  | MANF | PNN |
|  | ANKHD1 | ISG20L2 |  | RNH1 | GTF2F2 |
|  | PTPN2 | TTC13 |  | TWF2 | CCT6P1 |
|  | NFU1 | TPR |  | AKT1 | NFE2L2 |
|  | AKAP7 | SCAMP1 |  | AIP | NOL11 |
|  | ZNF337 | RAB22A |  | MCRS1 | GGPS1 |
|  | R3HCC1 | SNRPC |  | ETFA | BIRC2 |
|  | TAP2 | UBE2J1 |  | MCUB | ARL14EP |
|  | TTC19 | UBAC2 |  | PSMF1 | PTPN11 |
|  | XPOT | TIA1 |  | STX10 | MAP3K7 |
|  | YME1L1 | RASA1 |  | NDUFA4 | RAB8B |
|  | AP1G1 | RPF2 |  | BCL2L13 | SYNCRIP |
|  | ZNF700 | DHX40 |  | GTF2E2 | DHX29 |
|  | MARCHF6 | NCOA7 |  | TMEM203 | SRBD1 |
|  | C16orf72 | CYLD |  | DCTN3 | STK26 |
|  | NUP133 | VPS8 |  | RAB40C | C2CD5 |
|  | ANKRA2 | EIF4G3 |  | UBN1 | HNRNPH1 |
|  | RPRD1A | PNISR |  | BASP1 | MICU2 |
|  | OPTN | LARP7 |  | REPIN1 | TM2D1 |
|  | RAB3IP | SLF2 |  | RPA1 | KLRF1 |
|  | CENATAC | H2AJ |  | CLPTM1L | HMGCS1 |
|  | STX2 | CUL2 |  | VPS29 | MIS18BP1 |
|  | ITPR1 | ENSA |  | CHD4 | IL17RD |
|  | MPHOSPH8 | OXR1 |  | CYB561A3 | FAM160B1 |
|  | DCTN6 | FAM189B | | CCDC12 | PAG1 |
|  | COMMD10 | SKP2 |  | RHBDD2 | USO1 |
|  | ING3 | RABEP1 |  | POLD2 | ZRANB2 |
|  | ZSWIM6 | ARFGEF1 | | ATP6V1G1 | PSMA3 |
|  | TULP4 | PELI1 |  | SRF | FEM1C |
|  | SWAP70 | ZCCHC8 |  | GNPDA1 | DENND4C |
|  | ARPP19 | NIPBL |  | ELF4 | DHX36 |
|  | IARS1 | PHF20L1 |  | PLEKHM2 | UBE2Q2 |
|  | TSC22D2 | XPO4 |  | IMP4 | CEP350 |
|  | CRYZL1 | IL6ST |  | MRPL20 | EXO5 |
|  | CLASP1 | ZFAND1 |  | SEC61B | ZNF302 |
|  | RNF168 | PUM3 |  | MAT2B | FAM13B |
|  | CD58 | RCOR3 |  | CCDC32 | NUDT21 |
|  | SWI5 | DAP |  | ATP6AP2 | RPS6KA5 |
|  | YIPF6 | TRIM38 |  | JADE2 | CYB5R4 |
|  | MCCC1 | CNOT6 |  | NCL | STAG2 |
|  | LRRC8B | RAB27B |  | PDCD2 | SP3 |
|  | LRPPRC | SMC3 |  | SPTLC1 | EXOSC8 |
|  | UBE2K | FRYL |  | TSPAN17 | JKAMP |
|  | ICOS | CCSER2 |  | HMGN4 | RFC1 |
|  | ZNHIT6 | PIGB |  | BAX | NUP107 |
|  | DDX10 | FBXW7 |  | FAM120A | ZNF770 |
|  | APPL2 | MTERF3 |  | SAMM50 | DESI2 |
|  | FAM133B | ATP8B4 |  | CHMP4B | HTR2A |
|  | MFF | PCM1 |  | FYN | CMTR2 |
|  | MED23 | USP34 |  | FEM1A | TXK |
|  | MED8 | GSKIP |  | GPR137 | CCDC124 |
|  | CDKN2AIP | YIF1A |  | CZIB | CMPK1 |
|  | OTUD4 | RNF19A |  | UROS | THUMPD1 |
|  | AFG3L1P | U2SURP |  | PSMD6 | LTV1 |
|  | ASF1A | AHCTF1 |  | EDC4 | EIF3A |
|  | SEC23IP | FAM214A | | ARPC1A | SERINC1 |
|  | EPC1 | N4BP2L2 |  | PIK3R2 | TTC37 |
|  | ARID1B | CYSLTR1 | | KIAA0513 | NGLY1 |
|  | CDK2AP2 | CEP44 |  | NELFB | CSGALNACT2 |
|  | LIPT1 | ATG4C |  | MRPL11 | PCMTD1 |
|  | DNAJC13 | CNTRL |  | GMFG | GPR65 |
|  | BNIP3 | PPWD1 |  | NT5C | CHD1 |
|  | GNAQ | EPM2AIP1 | | RAE1 | PHIP |
|  | WDR7 | PHACTR2 | | SLC25A42 | SNX14 |
|  | SLC11A2 | MAP3K20 | | CAVIN2 | CCBE1 |
|  | CYP4V2 | DDX46 |  | MFSD1 | GNAI3 |
|  | ZNF518B | ACAP2 |  | CLPTM1 | AGL |
|  | TFB2M | CEP63 |  | DPP7 | ZNF559 |
|  | ACSL3 | MTMR9 |  | DMAC2 | HNRNPH3 |
|  | CLIP4 | CCNC |  | TRPV2 | SNX10 |
|  | PHF14 | RBM43 |  | PFDN1 | UBE4A |
|  | MTAP | UTP14C |  | ANKRD13A | SETDB2 |
|  | GOLM2 | HSPA14 |  | DNASE2 |  |
|  | PIM3 | RORA |  | EIF4EBP2 | |
|  | SFR1 | NRIP1 |  | SNRNP40 | |
|  | AP5M1 | NPHP3 |  | SEMA4B |  |
|  | UGGT1 | TAF2 |  | RHBDF2 |  |
|  | KIAA2026 | SAMSN1 |  | SSBP1 |  |
|  | NAPB | RIOK2 |  | NINJ2 |  |
|  | PDCD6IP | LTN1 |  | PRXL2A |  |
|  | GRPEL2 | ZNF800 |  | SELENOF | |
|  | LRATD2 | VCPKMT | | ZFP36L2 |  |
|  | WDR36 | ATXN3 |  | DPM3 |  |
|  | INSIG2 | GNPDA2 |  | EEF1AKNMT | |
|  | NDUFAF7 | XRN1 |  | MRPS11 |  |
|  | RAD23B | ZNF25 |  | AAR2 |  |
|  | IREB2 | SESTD1 |  | ALDOC |  |
|  | MYO9A | TIMM17B | | MRPL14 |  |
|  | AIM2 | ITCH |  | GNPTG |  |
|  | FNIP1 | FAR1 |  | RBX1 |  |
|  | PRMT3 | INO80D |  | MED20 |  |
|  | ARL1 | DDX60 |  | USP39 |  |
|  | MSANTD4 | CHUK |  | MRGBP |  |
|  | AP4E1 | HIBCH |  | SLC66A3 |  |
|  | UBR3 | PIGA |  | MRPS27 |  |
|  | METTL25 | SENP6 |  | DNAJA2 |  |
|  | TRMT1L | RWDD4 |  | FAM53C |  |
|  | SLFN5 | DENND1B | | STIP1 |  |
|  | IQCB1 | EXOC1 |  | L3MBTL2 | |
|  | KLHL3 | CDC14A |  | RNF4 |  |
|  | DNAH1 | TBC1D4 |  | GBA |  |
|  | MMUT | BAZ2B |  | UNC45A |  |
|  | DNM1L | REV1 |  | NUP93 |  |
|  | ACOX3 | CCNT2 |  | FOXN3 |  |
|  | SELENOT | NDUFB4 |  | SPHK2 |  |
|  | ZNF615 | CILK1 |  | CYBB |  |
|  | ZZZ3 | FBXO3 |  | NUP85 |  |
|  | ZNF17 | ZBTB20 |  | SLC9A1 |  |
|  | C11orf54 | EPS15 |  | ATG101 |  |
|  | LNPK | ZDHHC17 | | CENPX |  |
|  | RPAP2 | OPA1 |  | TOMM40 |  |
|  | WASL | SMAD5 |  | SLC50A1 |  |
|  | SNX1 | NFAT5 |  | DHPS |  |
|  | FAM122A | MTX3 |  | EMG1 |  |
|  | TSPYL2 | PON2 |  | SLFN11 |  |
|  | ETFDH | CWF19L2 | | PPP1R14B | |
|  | PCGF6 | SOCS4 |  | BUD23 |  |
|  | FAM122B | TRMT11 |  | TYSND1 |  |
|  | MIS12 | DNAJC27 | | DTX2 |  |
|  | PDE3B | JAK2 |  | C1orf50 |  |
|  | ZNF548 | TMF1 |  | MRPS21 |  |
|  | SLC35B3 | TRMT13 |  | SBF1 |  |
|  | SC5D | SEC24B |  | LDHB |  |
|  | POC5 | SUCLA2 |  | ADCK2 |  |
|  | GMCL1 | BIVM |  | CKS1B |  |
|  | CAPN3 | RRP15 |  | APBA3 |  |
|  | DMXL2 | MED15P9 | | GMPPA |  |
|  | CPEB3 | SLK |  | UBE2E1 |  |
|  | FAM126B | LCOR |  | RIPOR1 |  |
|  | KRAS | DCUN1D1 | | SET |  |
|  | CBR4 | SLC35A3 |  | NOTCH1 |  |
|  | MTM1 | ARMT1 |  | RPUSD2 |  |
|  | MIR600HG | SPOCD1 |  | SLC41A3 |  |
|  | MOSPD2 | USP8 |  | CFLAR |  |
|  | ZNF816 | THUMPD2 | | CYFIP1 |  |
|  | MRPS34 | TMX3 |  | GMDS |  |
|  | UBR5 | IBTK |  | PPIE |  |
|  | MED26 | ZNF654 |  | COX10 |  |
|  | IRAK4 | USP15 |  | PAFAH1B3 | |
|  | PAN2 | BMPR2 |  | UBE2S |  |
|  | NIPSNAP2 | SMC6 |  | HAUS7 |  |
|  | LEO1 |  |  | OSBPL5 |  |
|  | NHLRC3 |  |  | DCTPP1 |  |
|  | ANKRD17 | |  | PSD4 |  |
|  | CLASP2 |  |  | COPG1 |  |
|  | PI4K2B |  |  | LEPROTL1 | |
|  | VTA1 |  |  | ADORA2A | |
|  | AGPAT4 |  |  | IDS |  |
|  | ZSCAN16 | |  | SLC39A1 |  |
|  | STXBP5 |  |  | GDI2 |  |
|  | CHM |  |  | KARS1 |  |
|  | PRKAA1 |  |  | SNUPN |  |
|  | BTBD7 |  |  | BCAS4 |  |
|  | TCF12 |  |  | CDV3 |  |
|  | LOC100505715 | |  | SP2 |  |
|  | MAPK6 |  |  | SIGIRR |  |
|  | CETN3 |  |  | PRKAG1 |  |
|  | WDR19 |  |  | ACVR1B |  |
|  | MDFIC |  |  | CHCHD5 |  |
|  | ALDH5A1 | |  | WDR46 |  |
|  | CMSS1 |  |  | MAP3K3 |  |
|  | CEP135 |  |  | RPL23 |  |
|  | SND1-IT1 | |  | CENPBD1 | |
|  | ORC2 |  |  | NDST2 |  |
|  | CNOT6L |  |  | NUBP1 |  |
|  | RTCA |  |  | ZMAT5 |  |
|  | DAAM1 |  |  | GOLPH3 |  |
|  | YOD1 |  |  | SLC25A1 |  |
|  | EAF2 |  |  | NUAK2 |  |
|  | DNAJC24 | |  | UBQLN2 |  |
|  | WRAP53 |  |  | MORF4L1 | |
|  | NET1 |  |  | PEMT |  |
|  | GABPA |  |  | MRTFA |  |
|  | EHBP1 |  |  | BTN2A1 |  |
|  | RADX |  |  | WDR45B |  |
|  | ERO1A |  |  | FBXL15 |  |
|  | TTF1 |  |  | MAPK13 |  |
|  | USP14 |  |  | THOC5 |  |
|  | THAP1 |  |  | ACAT2 |  |
|  | PRMT7 |  |  | CDC42EP4 | |
|  | ZBTB24 |  |  | TANGO2 |  |
|  | GORAB |  |  | MADD |  |
|  | MYNN |  |  | WNK1 |  |
|  | ST8SIA4 |  |  | TESK1 |  |
|  | ZNF529 |  |  | EIF2D |  |
|  | ATF7IP2 |  |  | STK19 |  |
|  | FBXO5 |  |  | FEN1 |  |
|  | GFRA2 |  |  | MIEF1 |  |
|  | ERP27 |  |  | DECR1 |  |
|  | TBCK |  |  | DYNLL2 |  |
|  | EVI5 |  |  | NUDCD3 |  |
|  | AASDHPPT | |  | SSBP2 |  |
|  | ZNF189 |  |  | IKBKE |  |
|  | COG6 |  |  | THOC6 |  |
|  | PDE4D |  |  | BRF2 |  |
|  | SPAST |  |  | ILVBL |  |
|  | SPTY2D1 | |  | CD82 |  |
|  | ZWILCH |  |  | MED24 |  |
|  | NHLRC2 |  |  | ATP1B3 |  |
|  | TLR10 |  |  | NAIF1 |  |
|  | SLC25A24 | |  | UFM1 |  |
|  | ARFIP1 |  |  | LAP3 |  |
|  | RBBP9 |  |  | SPRYD3 |  |
|  | RB1 |  |  | PTGER4 |  |
|  | RPE |  |  | RUVBL1 |  |
|  | STAG1 |  |  | WDR45 |  |
|  | ATL2 |  |  | ZFAND3 |  |
|  | ZNF75D |  |  | LMNB2 |  |
|  | NDFIP1 |  |  | SH3BP5L |  |
|  | PTPN22 |  |  | COX15 |  |
|  | HIVEP1 |  |  | GPR132 |  |
|  | HELQ |  |  | MRPL12 |  |
|  | DCUN1D4 | |  | FAM174C | |
|  | BCAP29 |  |  | BICRA |  |
|  | ZBTB21 |  |  | TAB1 |  |
|  | ZNF260 |  |  | LETM1 |  |
|  | EXOC6 |  |  | MTIF3 |  |
|  | TXLNG |  |  | JADE1 |  |
|  | NEMP1 |  |  | VPS72 |  |
|  | ZMYM4 |  |  | INTS5 |  |
|  | TMEM30A | |  | MINDY1 |  |
|  | WRN |  |  | DAXX |  |
|  | SIRT1 |  |  | IL12RB1 |  |
|  | KRIT1 |  |  | TBC1D13 |  |
|  | TASOR |  |  | GMPR2 |  |
|  | ATP11B |  |  | SLC25A38 | |
|  | UTP23 |  |  | SNRNP27 | |
|  | PEX1 |  |  | SNAPC2 |  |
|  | KLHL20 |  |  | NLRX1 |  |
|  | GLCE |  |  | SHPK |  |
|  | AASDH |  |  | GALM |  |
|  | OSBPL3 |  |  | ASMTL |  |
|  | CSPP1 |  |  | PDRG1 |  |
|  | STK3 |  |  | LRP3 |  |
|  | GCNT1 |  |  | SIK3 |  |
|  | ZNF30 |  |  | XPO7 |  |
|  | PSIP1 |  |  | CYP1B1 |  |
|  | ZNF224 |  |  | TFPT |  |
|  | GAS2L1 |  |  | SLC35D2 |  |
|  | ANKRD50 | |  | ABHD14B | |
|  | STAU2 |  |  | HECTD3 |  |
|  | NEK7 |  |  | POLR2D |  |
|  | YTHDF3 |  |  | DNAJC7 |  |
|  | ATP6V1C1 | |  | GET4 |  |
|  | TLK1 |  |  | CASP9 |  |
|  | TWSG1 |  |  | ELOA |  |
|  | PRPF38B |  |  | SNX8 |  |
|  | EIF3J |  |  | GSS |  |
|  | CLK4 |  |  | PRCC |  |
|  | PLAA |  |  | DNAJC5 |  |
|  | GLMN |  |  | WSB1 |  |
|  | SCAPER |  |  | POP7 |  |
|  | ZFX |  |  | ELAC2 |  |
|  | EIF4E |  |  | HDGFL2 |  |
|  | SNX4 |  |  | SCAMP2 |  |
|  | CUL4B |  |  | EEF1E1 |  |
|  | ZNF439 |  |  | CASZ1 |  |
|  | UCK1 |  |  | BAD |  |
|  | MORC3 |  |  | RHBDD1 |  |
|  | ZUP1 |  |  | UQCR10 |  |
|  | SLC36A4 |  |  | LRRC28 |  |
|  | HAR1A |  |  | OGFOD3 |  |
|  | CST9 |  |  | GNGT2 |  |
|  | RPL29P2 |  |  | IMMT |  |
|  | PML |  |  | UBTD1 |  |
|  | WIPF2 |  |  | EVI5L |  |
|  | MEX3D |  |  | ZMYND19 | |
|  | TMEM164 | |  | TMEM159 | |
|  | PRUNE2 |  |  | TAF7 |  |
|  | IRAG1 |  |  | SLC2A8 |  |
|  | LINC02554 | |  | TMEM187 | |
|  | CASP8AP2 | |  | ELP5 |  |
|  |  |  |  | TTYH2 |  |
|  |  |  |  | ANKRD39 | |
|  |  |  |  | CYCS |  |
|  |  |  |  | MPDU1 |  |
|  |  |  |  | UQCRB |  |
|  |  |  |  | MOSPD3 |  |
|  |  |  |  | NOD1 |  |
|  |  |  |  | PEBP1 |  |
|  |  |  |  | GLOD4 |  |
|  |  |  |  | KLHL21 |  |
|  |  |  |  | ABHD12 |  |
|  |  |  |  | LRWD1 |  |
|  |  |  |  | DPM2 |  |
|  |  |  |  | CHST14 |  |
|  |  |  |  | ZNF282 |  |
|  |  |  |  | KLHL42 |  |
|  |  |  |  | PECR |  |
|  |  |  |  | PAQR4 |  |
|  |  |  |  | ADAM10 |  |
|  |  |  |  | FAM50B |  |
|  |  |  |  | SLC35F6 |  |
|  |  |  |  | VIPAS39 |  |
|  |  |  |  | INKA1 |  |
|  |  |  |  | B3GAT3 |  |
|  |  |  |  | PXMP2 |  |
|  |  |  |  | RAB5A |  |
|  |  |  |  | AP5S1 |  |
|  |  |  |  | THAP4 |  |
|  |  |  |  | CLIP2 |  |
|  |  |  |  | TUBG1 |  |
|  |  |  |  | PSMD5 |  |
|  |  |  |  | NUBP2 |  |
|  |  |  |  | NELFE |  |
|  |  |  |  | FLYWCH2 | |
|  |  |  |  | FAM170B | |
|  |  |  |  | TMEM9 |  |
|  |  |  |  | MRTO4 |  |
|  |  |  |  | TEX261 |  |
|  |  |  |  | PGAM5 |  |
|  |  |  |  | TK2 |  |
|  |  |  |  | SMIM5 |  |
|  |  |  |  | RAB33A |  |
|  |  |  |  | PHAX |  |
|  |  |  |  | MRPS18A | |
|  |  |  |  | LRRC8A |  |
|  |  |  |  | ACP4 |  |
|  |  |  |  | TTC9C |  |
|  |  |  |  | UCK2 |  |
|  |  |  |  | CD1A |  |
|  |  |  |  | KRCC1 |  |
|  |  |  |  | FZD7 |  |
|  |  |  |  | DOLK |  |
|  |  |  |  | BATF3 |  |
|  |  |  |  | CCDC28B | |
|  |  |  |  | RSRC2 |  |
|  |  |  |  | ZNF34 |  |
|  |  |  |  | TMEM40 |  |
|  |  |  |  | VAC14 |  |
|  |  |  |  | NRM |  |
|  |  |  |  | CALN1 |  |
|  |  |  |  | ATG14 |  |
|  |  |  |  | SPHK1 |  |
|  |  |  |  | ARSG |  |
|  |  |  |  | PAQR7 |  |
|  |  |  |  | SRP72 |  |
|  |  |  |  | TBL3 |  |
|  |  |  |  | TIMM9 |  |
|  |  |  |  | UQCRC2 |  |
|  |  |  |  | MRPL28 |  |
|  |  |  |  | NEXN |  |
|  |  |  |  | BAIAP2-DT | |
|  |  |  |  | PTPN9 |  |
|  |  |  |  | CHTF8 |  |
|  |  |  |  | SH2B2 |  |
|  |  |  |  | ABHD4 |  |
|  |  |  |  | BTD |  |
|  |  |  |  | CETP |  |
|  |  |  |  | ACOT2 |  |
|  |  |  |  | C1orf216 |  |
|  |  |  |  | ST3GAL2 | |
|  |  |  |  | FBXL20 |  |
|  |  |  |  | PINX1 |  |
|  |  |  |  | POLDIP2 |  |
|  |  |  |  | WFS1 |  |
|  |  |  |  | HYAL2 |  |
|  |  |  |  | FDX2 |  |
|  |  |  |  | MPND |  |
|  |  |  |  | DGCR6 |  |
|  |  |  |  | PXMP4 |  |
|  |  |  |  | TAF6 |  |
|  |  |  |  | CRAT |  |
|  |  |  |  | PTGIR |  |
|  |  |  |  | MUSK |  |
|  |  |  |  | DPCD |  |
|  |  |  |  | RCC1L |  |
|  |  |  |  | JMJD4 |  |
|  |  |  |  | PSPH |  |
|  |  |  |  | MAP3K6 |  |
|  |  |  |  | TMCO6 |  |
|  |  |  |  | IMPA1 |  |
|  |  |  |  | ZDHHC24 | |
|  |  |  |  | AGFG2 |  |
|  |  |  |  | ALKBH4 |  |
|  |  |  |  | AHSA2P |  |
|  |  |  |  | USP48 |  |
|  |  |  |  | FLCN |  |
|  |  |  |  | FAM98A |  |
|  |  |  |  | SHOC2 |  |
|  |  |  |  | MTF2 |  |
|  |  |  |  | CIR1 |  |
|  |  |  |  | GOLT1B |  |
|  |  |  |  | ATP2B1 |  |
|  |  |  |  | ZNF710 |  |
|  |  |  |  | UBXN8 |  |
|  |  |  |  | POLG2 |  |
|  |  |  |  | HHAT |  |
|  |  |  |  | C14orf178 | |
|  |  |  |  | CIB4 |  |
